# Projected Lifetime Cancer Risks From Current Computed Tomography Imaging

**DOI:** 10.1001/jamainternmed.2025.0505

**Published:** 2025-04-14

**Authors:** Rebecca Smith-Bindman, Philip W. Chu, Hana Azman Firdaus, Carly Stewart, Matthew Malekhedayat, Susan Alber, Wesley E. Bolch, Malini Mahendra, Amy Berrington de González, Diana L. Miglioretti

**Affiliations:** 1 Department of Epidemiology and Biostatistics, University of California, San Francisco; 2 Department of Obstetrics, Gynecology and Reproductive Sciences, University of California, San Francisco; 3 Philip R. Lee Institute for Health Policy Studies, University of California, San Francisco; 4 Department of Public Health Sciences, University of California, Davis; 5 J. Crayton Pruitt Family Department of Biomedical Engineering, University of Florida, Gainesville; 6 Division of Pediatric Critical Care, Department of Pediatrics, UCSF Benioff Children’s Hospital, University of California, San Francisco; 7 Division of Genetics and Epidemiology, The Institute of Cancer Research, London, United Kingdom; 8 Kaiser Permanente Washington Health Research Institute, Kaiser Permanente Washington, Seattle

## Abstract

**Importance:**

Approximately 93 million computed tomography (CT) examinations are performed on 62 million patients annually in the United States, and ionizing radiation from CT is a known carcinogen.

**Objective:**

To project the number of future lifetime cancers in the US population associated with CT imaging in 2023.

**Design, Setting, and Participants:**

This risk model used a multicenter sample of CT examinations prospectively assembled between January 2018 and December 2020 from the University of California San Francisco International CT Dose Registry. Data analysis was conducted from October 2023 to October 2024.

**Main Outcomes and Measures:**

Distributions of CT examinations and associated organ-specific radiation doses were estimated by patient age, sex, and CT category and scaled to the US population based on the number of examinations in 2023, quantified by the IMV national survey. Lifetime radiation-induced cancer incidence and 90% uncertainty limits (UL) were estimated by age, sex, and CT category using National Cancer Institute software based on the National Research Council’s Biological Effects of Ionizing Radiation VII models and projected to the US population using scaled examination counts.

**Results:**

An estimated 61 510 000 patients underwent 93 000 000 CT examinations in 2023, including 2 570 000 (4.2%) children, 58 940 000 (95.8%) adults, 32 600 000 (53.0%) female patients, and 28 910 000 (47.0%) male patients. Approximately 103 000 (90% UL, 96 400-109 500) radiation-induced cancers were projected to result from these examinations. Estimated radiation-induced cancer risks were higher in children and adolescents, yet higher CT utilization in adults accounted for most (93 000; 90% UL, 86 900-99 600 [91%]) radiation-induced cancers. The most common cancers were lung cancer (22 400 cases; 90% UL, 20 200-25 000 cases), colon cancer (8700 cases; 90% UL, 7800-9700 cases), leukemia (7900 cases; 90% UL, 6700-9500 cases), and bladder cancer (7100 cases, 90% UL, 6000-8500 cases) overall, while in female patients, breast was second most common (5700 cases; 90% UL, 5000-6500 cases). The largest number of cancers was projected to result from abdomen and pelvis CT in adults, reflecting 37 500 of 103 000 cancers (37%) and 30 million of 93 million CT examinations (32%), followed by chest CT (21 500 cancers [21%]; 20 million examinations [21%]). Estimates remained large over a variety of sensitivity analyses, which resulted in a range of 80 000 to 127 000 projected cancers across analyses.

**Conclusions and Relevance:**

This study found that at current utilization and radiation dose levels, CT examinations in 2023 were projected to result in approximately 103 000 future cancers over the course of the lifetime of exposed patients. If current practices persist, CT-associated cancer could eventually account for 5% of all new cancer diagnoses annually.

## Key Points

**Question:**

How many future cancers could result from radiation exposure from annual computed tomography (CT) examinations in the United States?

**Findings:**

In this risk model, the 93 million CT examinations performed in 62 million patients in 2023 were projected to result in approximately 103 000 future cancers. Although the per-examination cancer risk was higher in children, higher CT utilization among adults accounted for the majority of the projected cancers.

**Meaning:**

These findings suggest that if current radiation dosing and utilization practices continue, CT-associated cancers could eventually account for 5% of all new cancer diagnoses annually.

## Teaser

This risk model projects how many future cancers in the United States could result from annual computed tomography examinations.

## Introduction

Computed tomography (CT) is an indispensable and widely performed medical imaging test. Ongoing technological advancements expand its capabilities and popularity, and utilization continues to rise in the United States, exceeding prepandemic volume.^1^ While CT aids diagnosis, leading to improved outcomes, it also exposes patients to ionizing radiation at levels known to be associated with increased cancer risk. Several large retrospective cohort studies have shown that childhood exposure to CT is associated with increased risk of hematologic malignant neoplasms and brain cancer.^2,3,4,5^ In adults, cancer risks from low to moderate radiation doses are primarily based on studies of Japanese atomic bomb survivors or populations irradiated through medical or occupational exposures.^6,7^ However, there is also evidence that CT damages DNA in adults.^8^ Radiation-induced cancer risks from CT examinations vary by radiation dose, which depends on the clinical indication; body region imaged; patients’ sex, age, and size; and acquisition techniques.^9^ A 2009 analysis^10^ estimated that approximately 29 000 future cancers would result from routine CT exposures in the United States in 2007. The study authors used best-available data on the volume and distribution of examinations, approximations of radiation doses, and associated absorbed organ doses. Since then, the number of CT examinations performed annually in the United States has increased by more than 30%,^1^ more granular data have become available describing examination types, and more accurate methods have been developed for estimating organ dose.

This study updates previously projected lifetime cancer incidence associated with CT using the most recent utilization numbers available, empirical data on CT type by age and sex, and organ doses estimated directly from examination-level clinical data across the United States using best-practice methods. The purpose is to understand the public health impact of current CT use and to identify the highest risk examination types, age, and sex groups.

## Methods

This risk model used patient-level data from the University of California San Francisco (UCSF) International CT Dose Registry, which has assembled CT examinations from 143 US hospitals and outpatient facilities associated with 22 health care organizations in 20 states.^9^ For each examination, the registry captured Digital Imaging and Communications in Medicine (DICOM) metadata, including patient age, sex, effective diameter of the body part imaged, scanner type, examination name and description, and other technical acquisition parameters, such as kilovoltage, milliamperage, scan length, phase, pitch, and collimation. The UCSF Committee on Human Research approved the study with a waiver of consent due to the large number of records making it impractical to contact all participants, the researchers not knowing the identity of the participants; and the risk of contacting participants being greater than the study risks. Collaborating institutions obtained local ethics approval. We have followed the Strengthening the Reporting of Observational Studies in Epidemiology (STROBE) reporting guideline.

### CT Utilization

We used the IMV Medical Information Division CT Market Outlook Report, based on a national, annual survey of 235 hospitals and 78 imaging facilities, to quantify the number of CT examinations performed in the United States in 2023.^1^ IMV medical imaging utilization data have been validated against sources such as Medicare and the Veterans Administration by the US National Council on Radiation Protection Report No. 184 and used in several publications.^11,12^ To apportion examinations between adults and children, we used the proportion of pediatric examinations in 2022 in the American College of Radiology (ACR) National Radiology Data Registry (Judy Burleson, MHSA, and Mike Simanowith, MD, ACR, email, November 13, 2023). To estimate the number of patients who underwent CT in 2023, longitudinal data from the registry from 2016 and 2020 were used to estimate the annual number of examinations per patient by age and sex (mean ranged from 1.1-1.7). This average was applied to the total number of CT examinations in 2023, by sex and age group, to estimate the number of patients exposed.

### Distribution of Examinations by Age, Sex, and CT Category

Using DICOM metadata, CT examinations in the registry were assigned to 1 of 26 CT categories that reflect a combination of body region and clinical indication (18 in adults^13^; 13 in children [Denise Bos, MD, unpublished data, March 2025) (eMethods in Supplement 1). Some CT categories represent a single body region (eg, cervical spine), while other regions are subdivided into categories reflecting radiation dose needs of the underlying indication (eg, in the abdomen, low dose includes imaging for kidney stones, routine dose for trauma, and high dose for cancer).

To estimate distributions of scans by age, sex, and CT category, we used pediatric examinations (ages 0-17 years) from the registry from January 2018 to December 2020 (46 559 patients) and adult examinations (ages 18-99 years) from January to December 2020 (74 653 patients). We excluded CT examinations associated with biopsies and procedures, positron emission tomography, or research (all infrequent) as well as age, sex, and CT category strata with fewer than 12 examinations given that estimated doses could be imprecise. Additional years of data were used for pediatric examinations (2018-2020) compared with adult examinations (2020) to ensure stable estimates within strata. We verified that the distribution of CT categories and radiation dose per category remained stable in adults from 2018 to 2020. From this sample of 121 212 examinations, we estimated the proportions of examinations by age, sex, and CT category resulting in 418 strata: 288 in adults (18 CT categories, 8 age groups, and 2 sexes); and 130 in children (13 CT categories, 5 age groups, 2 sexes).

### Individual Patient–Dependent Organ Dose Reconstruction

We estimated absorbed doses (radiation transport code MCNPX version 2.70 [Los Alamos National Laboratory]) for 18 organs for each CT examination through Monte Carlo radiation transport simulations using exact, examination-level technical parameters and patient size mapped to morphometry-matched hybrid computational phantoms from the University of Florida/National Cancer Institute phantom library.^14,15,16,17^ We then calculated mean organ doses (and SDs) in milliGray (mGy) for each strata.

### Statistical Analysis

#### Cancer Risk Estimation

We projected future lifetime radiation-induced cancer risk using the National Cancer Institute’s Radiation Risk Assessment Tool (RadRAT)^18,19^ software version 4.3.1, which utilizes risk models from the National Academy of Sciences’ Biologic Effects of Ionizing Radiation (BEIR) VII report for 11 site-specific cancers (stomach, colon, liver, lung, breast, uterus, ovary, prostate, bladder, and thyroid cancer and leukemia), plus 7 additional cancer sites (oral cavity or pharynx, esophagus, rectum, pancreas, kidney, and brain or central nervous system cancer plus a remainder category) using a more recent follow-up of the Japanese atomic bomb survivors and pooled analyses of other medically exposed cohorts.^18^ For a given cancer type, RadRAT estimates excess lifetime risk of cancer from the time of exposure based on user-supplied organ dose and US life table estimates of age- and sex-specific baseline cancer rates. These risk estimates account for death as a competing risk using sex-specific life table estimates for the US 2019 population. We developed solutions to expedite bulk use of RadRAT to estimate risks within the 418 strata (eMethods in Supplement 1).

#### Cancer Projections

We scaled the registry-based distribution of CT categories by age and sex by the IMV-derived total number of examinations, using the ACR percentage of pediatric examinations, to estimate the distribution of examinations at the US population level in 2023. We excluded examinations that occurred in the last year of life, which are unlikely to contribute to future cancers given the average latency between CT exposure and radiation-induced cancer development. To determine this proportion, we quantified the number of CT examinations performed in 2022 in patients’ last 1 and 2 years of life for each strata of age and sex at Kaiser Permanente Northern California, following published methods.^19,20^ Overall, 10.6% of scans were performed in the last year of life (9.4% in female patients and 12.1% in male patients), varying from 0.9% in children ages 1 to 4 years (1.4% girls and 0.5% boys) to 38.6% in adults ages 90 to 99 years (35.1% women and 44.4% men). We then applied the projected cancer rates from RadRAT to nationally scaled examination counts (reduced by the proportion of end-of-life examinations) to estimate lifetime cancer incidence and 90% uncertainty limits (UL) resulting from CT examinations in 2023. Since future cancer estimates are based on a linear model of the total radiation dose received, the projected number of cancers remains the same regardless of whether the analysis is based on the number of patients (62 million, who each underwent an average of 1.5 scans) or examinations (93 million).

#### Uncertainty Estimates and Sensitivity Considerations

RadRAT uses Monte Carlo simulation based on Latin hypercube sampling to account for uncertainty in the radiation risk model coefficients, transfer of risks from the Japanese to the US population, the dose and dose rate reduction effectiveness factor (DDREF), uncertainty in organ doses, and adjustments to minimal latency periods.^18^ A latency adjustment was phased in between 4.0 and 11.0 years after exposure for solid cancers, 0.4 and 4.1 years for leukemia, and 2.5 and 7.6 years for thyroid cancer. To represent uncertainty in the adjustments for minimum latency on risk estimates, the midpoint, μ, is described by the following triangular probability distributions: solid cancers other than thyroid, T(5, 7.5, 10); thyroid, T(3, 5, 7); and leukemia, T(2, 2.25, 2.5), where numbers represent time after exposure in years. RadRAT outputs 90% ULs, providing an upper and lower estimate of potential future cancers.

Sensitivity analyses were conducted modifying the baseline model assumptions. First, we applied male lung cancer risk coefficients to female patients in our projections because some epidemiological studies have not supported the 3-fold higher risk of radiation-induced lung cancer in female compared with male patients in BEIR VII.^21^ Second, we reduced the estimated annual imaging volume by 10% to account for potential overestimation by IMV, and third, we increased it by 10% for potential underestimation. Fourth, we reduced organ doses by 20% to allow for possible national differences from the UCSF registry, and fifth, we increased them by 20%. Sixth, we applied the higher IMV estimate of the percentage of CT examinations in children (9.0% vs 3.3%). Seventh, we used the distribution of examinations by age and CT category from calendar years 2018 to 2019, in case the 2020 distribution was atypical due to COVID-19. Last, we excluded CT examinations performed in the last 2 years, rather than 1 year, of life, varying by age and sex. All analyses used SAS version 9.3 (SAS Institute) and R version 4.2.2 (2022-10-31 ucrt [R Project for Statistical Computing]). Data analysis was conducted from October 2023 to October 2024.

## Results

**Figure 1.**
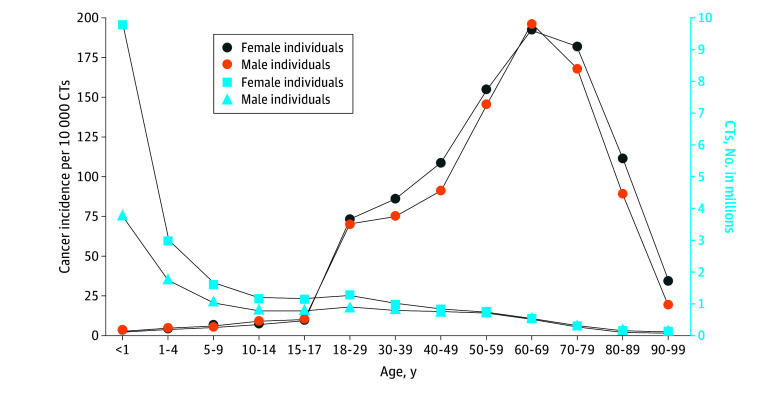
Number of Computed Tomography (CT) Examinations and Cancer Incidence by Sex The projected number of future cancers was estimated using the reduced number of CT examinations (excluding examinations that occur in the last year of life) as reported in Table 2. Cancer incidence (left axis, light blue circles and triangles) was based on the total number of examinations (right axis; dark blue and orange circles), a conservative estimate.

Ninety-three million CT examinations were performed in 61 510 000 patients in the United States in 2023, including an estimated 3 069 000 CTs (3.3%) in 2 570 000 children (4.2%) and 89 931 000 CTs (96.7%) in 58 940 000 adults (95.8%) (Table 1; eTable 1 in Supplement 1). Patients underwent a mean of 1.5 examinations each, varying by age (Table 1), and the median number of examinations per patient was 1 across all age groups. The total number of examinations increased with age for all CT categories, peaking in adults ages 60 to 69 years (Figure 1; eTable 1 in Supplement 1). After excluding examinations performed in the last year of life, a total of 84 161 000 were included for estimating cancer risks.

**Table 1.** Estimated Number of Patients Who Underwent CT and Number of CT Examinations in the United States in 2023, by Sex, Age, and Body Region^a^

Age, y	Patients, No.	CT examinations, No. (%)	Examinations per patient, mean, No.^b^	CT examination type, No.							
				Abdomen and pelvis	Head	Chest	Spine	Head and neck combined	Cardiac	Full body^c^	Extremities^d^
**Total population**											
All	61 510 000	93 000 000 (100)	1.51	30 221 400	24 115 800	19 975 300	7 735 200	2 826 900	1 073 900	4 607 000	2 444 500
Child	2 570 000	3 069 000 (3.3)	1.19	689 900	1 600 800	275 200	290 000	NA	26 400	57 300	129 300
Adult	58 940 000	89 931 000 (96.7)	1.53	29 531 500	22 515 000	19 700 100	7 445 200	2 826 900	1 047 500	4 549 700	2 315 200
**Female**											
All ages	32 600 000	48 549 200 (52.2)	1.49	16 669 900	12 263 700	10 136 700	4 082 800	1 494 100	513 800	2 228 000	1 160 200
<1	90 000	97 000 (0.1)	1.12	4000	72 200	13 100	3100	NA	4500	0	0
1-9	370 000	439 300 (0.5)	1.18	83 100	255 800	43 300	38 500	NA	3200	7900	7400
10-17	740 000	870 000 (0.9)	1.18	270 600	370 500	73 100	93 500	NA	4000	14 500	43 900
18-39	5 960 000	7 972 500 (8.6)	1.34	3 676 800	1 948 000	1 117 300	651 300	190 200	0	219 100	169 800
40-59	8 980 000	13 147 100 (14.1)	1.46	5 136 000	2 953 300	2 590 900	1 004 100	328 700	244 400	589 900	299 800
60-79	12 020 000	18 767 100 (20.2)	1.56	5 546 600	4 290 900	5 010 800	1 600 000	504 500	237 200	1 059 500	517 700
80-99	4 440 000	7 256 200 (7.8)	1.63	1 952 800	2 373 000	1 288 200	692 300	470 700	20 500	337 100	121 600
**Male**											
All ages	28 910 000	44 450 900 (47.8)	1.54	13 551 400	11 852 300	9 838 700	3 652 500	1 332 800	560 100	2 379 100	1 284 500
<1	100 000	119 800 (0.1)	1.14	6300	87 000	17 400	2200	NA	5900	1100	0
1-9	490 000	580 100 (0.6)	1.18	108 700	356 400	45 200	48 900	NA	3500	10 800	6700
10-17	780 000	962 800 (1.0)	1.23	217 100	459 000	83 100	103 800	NA	5300	23 100	7400
18-39	5 160 000	7 282 700 (7.8)	1.41	2 501 800	2 070 800	909 000	754 900	278 100	59 000	344 300	364 800
40-59	7 770 000	11 780 600 (12.7)	1.52	3 928 500	2 872 600	2 458 500	931 900	304 600	249 200	645 300	390 100
60-79	11 320 000	18 278 300 (19.7)	1.61	5 414 100	4 327 000	5 080 600	1 336 400	456 300	237 200	1 059 500	367 200
80-99	3 290 000	5 446 600 (5.9)	1.66	1 374 900	1 679 500	1 244 900	474 400	293 800	0	295 000	84 300

Abbreviations: CT, computed tomography; NA, not applicable, meaning this category does not exist in this age group.

^a^ More granular results by CT category within each body region are in eTable 1 in Supplement 1. Row values may not sum to “CT examinations” column due to rounding.

^b^ These are the actual calculated means based on the exact number of patients.

^c^ Full body includes whole body examinations in children and combined chest, abdomen, and pelvis examinations in adults.

^d^ Extremities includes all upper and lower extremity examinations.

### Organ Doses

Organ doses by body regions and sex are shown for sample age strata (eTable 2 in Supplement 1). Doses were similar but not identical by sex for most categories. For example, the mean (SD) brain dose for routine-dose head CT in children ages 5 to 9 years was 5% higher in boys (48.0 [27.3] mGy) than in girls (45.7 [24.1] mGy). Other categories, such as full body, had larger differences. For example, there was a 29% increase in pancreas dose between boys aged 5 to 9 years (21.5 [13.5] mGy) vs girls aged 5 to 9 years (16.7 [8.9] mGy). In general, organ doses were similar in children and adults or increased with age. For example, the mean (SD) colon dose in routine abdomen and pelvis CT was approximately twice as high in women aged 50 to 59 years (25.4 [15.2] mGy) vs girls aged 5 to 9 years (12.8 [8.7] mGy). However, there were exceptions: organ doses were highest overall in children younger than 1 year (eg, mean [SD] brain dose for routine head CT in boys <1 year was 60.0 [36.5] mGy), and mean (SD) bone marrow doses in head CT decreased with age (eg, boys <1 year, 26.7 [16.7] mGy; boys aged 5-9 years, 14.6 [10.0] mGy; and men aged 50-59 years, 3.5 [2.7] mGy).

### Projected Cancer Risks

CT utilization in the United States in 2023 was estimated to result in 102 700 (90% UL, 96 400-109 500) projected lifetime cancers, including 93 000 (90% UL, 86 900-99 600) in adults and 9700 (90% UL, 8100-11 600) in children (Table 2). The leading cancers in adults were lung cancer (21 400 [90% UL, 19 200-24 000]), colon cancer (8400 [90% UL, 7500-9400]), and leukemia (7400 [90% UL, 6100-8900]), whereas the most frequent projected cancers in children were thyroid (3500 [90% UL, 2300-5500]), lung (990 [90% UL, 870-1100]), and breast (630 [90% UL, 550-730]) cancer (Table 2; eFigure in Supplement 1). Lung and thyroid cancer incidence were higher in female patients, whereas incidence of most other cancers was similar by sex or slightly higher in male patients (eFigure and eTable 3 in Supplement 1).

**Table 2.** Projected Number of Future Cancers Overall and by Cancer Type Associated With CT Examinations Performed in the United States in 2023, by Age Group and Body Region^a^

Cancer type	Projected future cancers, No. (90% UL)		
	All CT examinations (N = 93 000 000)	CT examinations in adults (n = 89 931 000)	CT examinations in children (n = 3 069 000)
Total	102 700 (96 400-109 500)	93 000 (86 900-99 600)	9700 (8100-11 600)
Projected cancer by type			
Lung	22 400 (20 200-25 000)	21 400 (19 200-24 000)	990 (870-1100)
Colon	8700 (7800-9700)	8400 (7500-9400)	330 (280-390)
Leukemia	7900 (6700-9500)	7400 (6100-8900)	550 (380-820)
Bladder	7100 (6000-8500)	6900 (5700-8200)	250 (200-320)
Stomach	7100 (5500-9100)	6800 (5200-8800)	280 (200-400)
Thyroid	7000 (5400-9200)	3500 (2700-4600)	3500 (2300-5500)
Breast	5700 (5000-6500)	5100 (4400-5900)	630 (550-730)
Liver	4100 (3400-5000)	4000 (3200-4900)	160 (130-200)
Kidney	3000 (2300-3900)	2900 (2200-3700)	130 (90-180)
Pancreas	2800 (2300-3500)	2700 (2200-3400)	100 (80-140)
Oral cavity or pharynx	2800 (2300-3400)	2300 (1900-2900)	450 (310-650)
Brain or CNS	1600 (1300-2000)	1200 (910-1500)	440 (320-620)
Esophagus	1500 (1300-1800)	1400 (1200-1700)	110 (90-150)
Prostate	1500 (820-2700)	1400 (760-2700)	70 (30-170)
Ovary	890 (670-1200)	850 (630-1100)	40 (30-70)
Rectum	560 (480-660)	540 (450-630)	30 (20-40)
Uterus	550 (400-760)	530 (380-730)	30 (16-50)
Other and ill-defined sites	17 400 (15 300-19 800)	15 800 (13 700-18 200)	1600 (1200-2000)
Projected cancer by CT examination body region			
Abdomen and pelvis	39 100 (34 600-44 200)	37 500 (32 900-42 600)	1600 (1300-2000)
Chest	22 700 (19 600-26 300)	21 500 (18 400-25 200)	1200 (960-1400)
Spine	12 900 (11 500-14 500)	11 600 (10 200-13 200)	1300 (1000-1600)
Head	12 500 (10 600-14 700)	7300 (6200-8700)	5100 (3700-7100)
Full body	8000 (7000-9100)	7600 (6600-8800)	320 (260-390)
Head and neck combined	4100 (3500-4800)	4100 (3500-4800)	NA
Cardiac	3400 (3200-3700)	3300 (3000-3600)	170 (140-210)
Extremity	80 (60-90)	70 (50-80)	9 (7-11)

Abbreviations: CNS, central nervous system; CT, computed tomography; NA, not applicable, meaning this category does not exist in this age group; UL, uncertainty limit.

^a^ More granular results by sex and cross-tabulation by body region and cancer type appear in eTable 3 in Supplement 1.

#### Projected Cancers by Age

**Figure 2.**
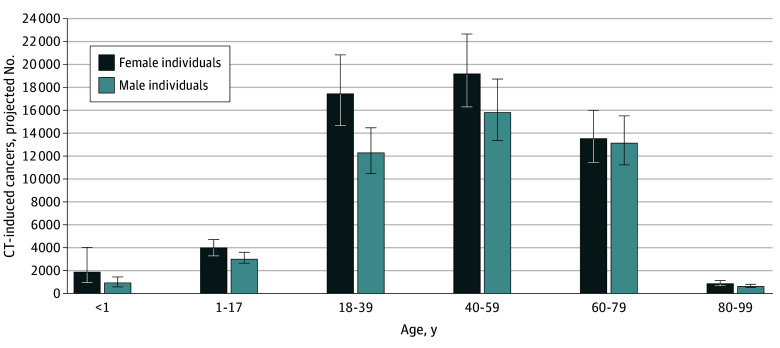
Total Projected Lifetime Cancers by Sex and Age at Exposure Error bars represent 90% uncertainty limits. CT indicates computed tomography.

Projected cancer risks per CT examination were estimated to be highest among children who underwent CT at younger than 1 year and decreased with age at exposure (Figure 1). For example, cancer risk in girls younger than 1 year were 20 cancers per 1000 examinations (1900 of 97 000) versus 2 per 1000 in girls aged 15 to 17 years (1100 of 483 600) (eTables 1 and 4 in Supplement 1). However, despite the higher risk per examination in children, higher utilization contributed to more projected cancers in adults (Table 2 and Figure 2). CT use in adults aged 50 to 59 years was associated with the highest number of projected cancers: 10 400 (90% UL, 8200-13 000) in female patients and 9300 (90% UL, 7500-11 700) in male patients (eTable 4 in Supplement 1).

#### Projected Cancers by CT Category

**Figure 3.**
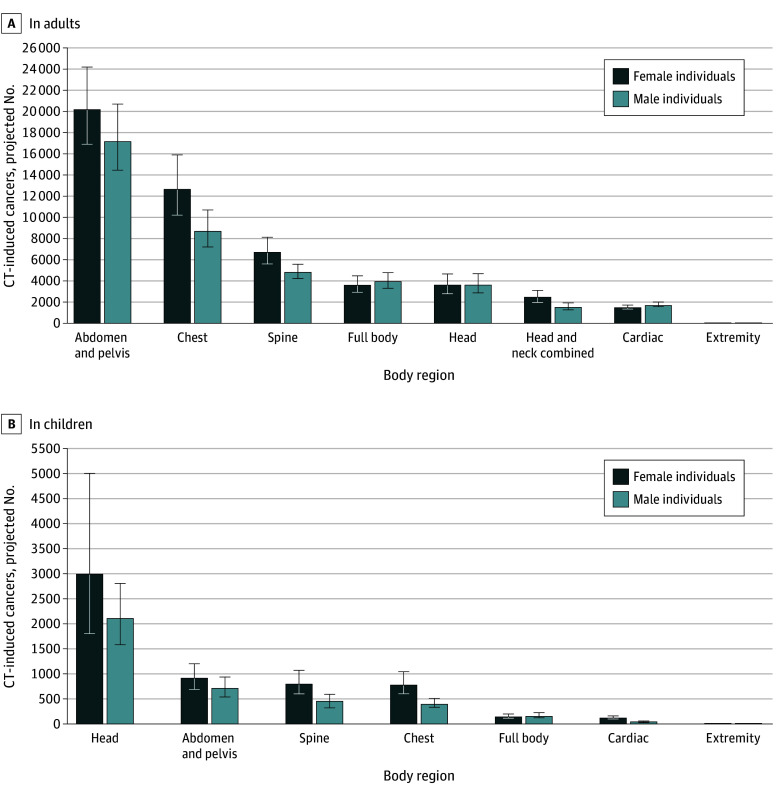
Projected Number of Computed Tomography (CT)–Induced Cancers by Body Region Imaged in Adults and Children, by Sex Error bars represent 90% uncertainty limits.

Abdomen and pelvis CT was estimated to contribute the largest number of projected cancers (40%) in adults (37 500 [90% UL, 32 900-42 600] cases), whereas head CT contributed the largest number of cancers (53%) in children (5100 [90% UL, 3700-7100) (Table 2 and Figure 3; eTable 3 in Supplement 1). For a few categories, such as full body, the projected proportion of cancers (8000 [7.8%]) was greater than the proportion of scans (4 607 000 scans [5.0%]) (Tables 1 and 2).

#### Sensitivity Analyses

The sensitivity analyses generated a range in estimated future cancers from 79 900 to 126 600 by reducing and increasing organ doses by 20%, respectively, reflecting 22.2% fewer cancers to as many as 23.3% more cancers than the primary analysis (eTable 6 in Supplement 1). Using the IMV-estimated proportion of pediatric examinations resulted in an 11.0% increase in projected cancers overall (to 114 000) and an increase in the proportion of cancers from childhood imaging from 9.4% to 23.2%.

## Discussion

CT is frequently lifesaving, yet its potential harms are often overlooked, and even very small cancer risks will lead to a significant number of future cancers given the tremendous volume of CT use in the United States. For current utilization and radiation dosing practices, we projected approximately 103 000 future cancers could result from CT use in the United States in 2023 (with sensitivity analyses projecting a range of 80 000 to 127 000) among the 62 million people who underwent CT. To provide context, if the number of new cancer diagnoses in the United States remains stable (1.95 million in 2023) and both the utilization and radiation doses from CT remain unchanged in future decades, CT could be responsible for approximately 5% of cancers diagnosed each year. This would place CT on par with other significant risk factors, such as alcohol consumption (5.4%) and excess body weight (7.6%).^22^

The projected number of radiation-induced cancers in this analysis is 3 to 4 times higher than the earlier assessment of CT exposure for several reasons.^10^ First, while growth in utilization has slowed over the intervening years,^20^ CT use is 30% higher today than in 2007, due to growth in low-value, potentially unnecessary imaging^23,24,25,26,27^ as well as population aging. Second, dose modeling in this study accounted for multiphase scanning, which occurs in 28.5% of examinations but was not modeled in the prior study, as multiphase frequency was unknown. Third, the substantially higher organ doses in this study were reconstructed using newer dosimetry methods with examination-level data from more than 120 000 actual examinations, while the prior study modeled doses from national survey data or imaging protocols and assumed examinations in children were performed using pediatric-specific settings. Lastly, we included more granular CT categories reflecting imaging indications that have important dose differences. Both studies used the same BEIR VII risk models; thus, this would not explain the large observed differences.

Lung cancer was projected to be the most common radiation-induced cancer, with 22 400 cases (eTable 4 in Supplement 1). Approximately 70% of these were in female patients, reflecting the higher BEIR VII risk coefficients in female patients. However, even when we applied male risk coefficients for female examinations, lung cancers were still the most common radiation-induced cancer in female patients. Colon cancer was the next most common, with 8700 cases (58.6% in males). It is unclear whether the current, unexplained increase in these 2 cancers as well as others at unexpectedly younger ages^28^ may be partly due to CT. Thyroid cancer also revealed notable sex differences, likely due to risk coefficients. For example, 1400 vs 320 thyroid cancers were projected to result from CT exposure in female and male patients, respectively, performed when the patient was younger than 1 year, despite equal thyroid organ doses (74.4 and 75.2 mGy) (eTables 2 and 4 in Supplement 1) and more examinations in male patients (Table 1). Our estimates from childhood CT exposure are higher than those in the large EPI-CT cohort study of pediatric cancer outcomes^2,3^ because we estimated lifetime risk of all cancer types, while EPI-CT examined brain and hematologic cancers within 15 years after exposure.

Abdomen and pelvis CT were projected to cause the greatest number of cancers. These and other examination types, such as high-dose abdomen and pelvis, full body, and spine CT, incur greater risks on average per examination because they frequently use multiple scan phases that result in higher doses.^29,30^ Often these examinations could use single-phase scanning, which would lower doses without impacting diagnostic accuracy.

This study estimated future cancers using the National Academy of Science BEIR VII–based modeling approach, which is widely accepted in the field of radiation epidemiology. While observational studies have directly quantified childhood cancer risk related to pediatric CT,^2,3,4,5^ for adult exposures, direct estimates are currently unavailable. To empirically quantify lifetime risk would require decades-long follow-up studies of very large populations, as the Life Span Study has done in Japanese bombing survivors. Thus, to feasibly capture full lifetime risk requires a modeling approach, and there is increasing evidence of elevated cancer risks from other low-dose radiation research supporting these risk estimates.^6,7,31^ While the BEIR VII cancer risk models are the most widely used and accepted approach for quantifying the cancer risks from low-dose radiation, several other studies have published risk models, such as the US Environmental Protection Agency, the UK National Radiological Protection Board, and the United Nations Scientific Committee on the Health Effects of Atomic Radiation (UNSCEAR). The risk estimates from these studies are broadly consistent with BEIR VII as well as estimates from the CT study cohorts including EPI-CT.^32^

Many of the model assumptions were conservative. For example, we used the ACR’s percentage of estimated examinations in children, which is lower than the percentage from IMV. We did not include CT-guided procedures, such as biopsies, which often use higher doses. Furthermore, it is possible that the low-energy x-rays emitted by CT scans cause more cellular damage compared with gamma rays, which were the primary source of radiation released from the atomic bombs.^33^ Lastly, RadRAT applied the DDREF of 1.5 (90% uncertainty interval, 1.1-2.3) recommended by BEIR VII to account for differences between low-dose exposure and the higher doses for which the models were developed. This assumes lower radiation doses are less harmful (per unit) than higher doses, based on the BEIR VII estimate that the risk of solid cancer per unit of radiation dose may be 1.5 times lower for doses of 100 mGy or less.^6^ However, several systematic reviews of low dose (<100 mGy) and low dose rate exposures support a DDREF of 1.^34,35^ If accurate, the true number of projected cancers would be closer to the upper end of the sensitivity estimates than the primary analysis projects.

### Strengths and Limitations

This study has several strengths, including detailed data on CT utilization and associated radiation dose, detailed calculation of risks with uncertainty limits, and sensitivity analyses that provide a range of estimates under widely varying assumptions. There are several limitations: first, the BEIR VII risk estimated model parameters are based primarily on the Japanese survivor outcomes, and questions remain about the transfer of radiation risks from the mid-20th century Japanese population to the current US population. The use of a weighted average of the excess relative and excess absolute risk models aims to partly account for this, but these weights are subjective.^36^ Second, our risk calculations factored in average life expectancies, and the degree to which patients who undergo CT have shorter life expectancy due to underlying illness may overestimate future cancer risk. However, we excluded on average 10.6% of CTs that were likely performed during the last year of life, given these patients are not at risk of a radiation-induced cancer. A recent analysis found that 9.6% of patients who undergo CT died within 1 year,^37^ similar to our estimate. Third, while the CT categorization algorithm was 90% accurate compared with expert review,^13^ some examinations in the registry may have been miscategorized; however, this is unlikely to significantly impact our results.

## Conclusions

In this study, approximately 5% of annual cancer diagnoses or 100 000 cancers were projected to result from CT utilization in 2023. Despite public attention to the potential adverse effects, CT use has grown significantly in the United States since 2009. In 2023, 93 million CT examinations were performed in the United States; in 2007, the number was 68.7 million—a 35% increase incompletely explained by population growth.^38^ Justification of use and optimization of dose, including consideration of the need for multiphase examinations, are the tenets of CT imaging and must be applied uncompromisingly to mitigate potential harm.

## Notes

Supplement 1.
eMethods.
**eTable 1.** Estimated number of computed tomography examinations performed in the US in 2023, by age, sex, and CT category **eTable 2.** Sample computed tomography organ doses (mean, standard deviation) by CT category and sex, for exposures at age <1, 5-9 years, and 50-59 years. The CT category Lower Extremity, high dose was not represented in children <1 year of age or 5-9 years of age and is thus not included. **eTable 3.** Projected number of future cancers (with uncertainty limits, UL) overall and by cancer type that could result from computed tomography examinations performed in the US in 2023, by body region, age group (adults vs children) and sex **eTable 4.** Projected number of future cancers (with uncertainty limits, UL) overall and by cancer type that could result from computed tomography examinations performed in the US in 2023, by age and sex **eTable 5.** Projected number of future cancers (with uncertainty limits, UL) by CT category, age and sex that could result from computed tomography examinations performed in the US in 2023 **eTable 6.** Projected number of cancers (with uncertainty limits, UL) in the US for each sensitivity analysis changing the baseline model assumptions **eFigure.** Projected number of CT-induced cancers by cancer type in adults (eFigure 1A) and children (eFigure 1B), by sex

Supplement 2.
Data Sharing Statement
