# Supplementary material for: Projected Lifetime Cancer Risks From Current Computed Tomography Imaging
Source: JAMA Intern Med. 2025 Apr 14;185(6):710–9. doi: 10.1001/jamainternmed.2025.0505 (PMC11997853; doi:10.1001/jamainternmed.2025.0505)
Supplement: Supplement 1. — eMethods. eTable 1. Estimated number of computed tomography examinations performed in the US in 2023, by age, sex, and CT category eTable 2. Sample computed tomography organ doses (mean, standard deviation) by CT category and sex, for exposures at age <1, 5-9 years, and 50-59 years. The CT category Lower Extremity, high dose was not represented in children <1 year of age or 5-9 years of age and is thus not included. eTable 3. Projected number of future cancers (with uncertainty limits, UL) overall and by cancer type that could result from computed tomography examinations performed in the US in 2023, by body region, age group (adults vs children) and sex eTable 4. Projected number of future cancers (with uncertainty limits, UL) overall and by cancer type that could result from computed tomography examinations performed in the US in 2023, by age and sex eTable 5. Projected number of future cancers (with uncertainty limits, UL) by CT category, age and sex that could result from computed tomography examinations performed in the US in 2023 eTable 6. Projected number of cancers (with uncertainty limits, UL) in the US for each sensitivity analysis changing the baseline model assumptions eFigure. Projected number of CT-induced cancers by cancer type in adults (eFigure 1A) and children (eFigure 1B), by sex [file jamainternmed-e250505-s001.pdf]

Supplemental Online Content

Smith-Bindman R, Chu PW, Firdaus HA, et al. Projected lifetime cancer risks from current computed tomography imaging. *JAMA Intern Med*. Published online April 14, 2025. doi:10.1001/jamainternmed.2025.0505

eMethods.

eTable 1. Estimated number of computed tomography examinations performed in the U.S. in 2023, by age, sex, and CT category.

eTable 2. Sample computed tomography organ doses in mGy (mean, standard deviation) by CT category and sex, for exposures at age <1, 5-9 years, and 50-59 years. CT categories that were not represented in children are not shown.

eTable 3. Projected number of future cancers (with uncertainty limits, UL) overall and by cancer type that could result from computed tomography exams performed in the U.S. in 2023, by body region, age group (adults vs children) and sex.

eTable 4. Projected number of future cancers (with uncertainty limits, UL) overall and by cancer type that could result from computed tomography exams performed in the U.S. in 2023, by age and sex.

eTable 5. Projected number of future cancers (with uncertainty limits, UL) by CT category, age and sex that could result from computed tomography exams performed in the U.S. in 2023.

eTable 6. Projected number of cancers (with uncertainty limits, UL) in the U.S. for each sensitivity analysis changing the baseline model assumptions.

eFigure. Projected number of CT-induced cancers and 90% uncertainty limits by cancer type in adults (eFigure 1A) and children (eFigure 1B), by sex.

This supplemental material has been provided by the authors to give readers additional information about their work.

© 2025 Smith-Bindman R et al. *JAMA Internal Medicine.*

eMethods

Using RadRAT to calculate risks to a population

The National Cancer Institute's Radiation Risk Assessment Tool (RadRAT) software was designed to calculate lifetime risk for a single person, which presents a challenge to calculating risk for a population. To achieve this goal, we developed the following workaround.

1. Synthetic patients

We first created “synthetic patients” representing each of the 418 strata (18 CT categories, 8 age groups, 2 sexes in adults; 13 CT categories, 5 age groups, 2 sexes in children). We used the mean organ doses for the CT category/age group/sex strata, and the mid-point of the age group for the synthetic patient’s age (e.g. age 35 years for a synthetic patient representing ages 30-39 years).

2. Batch upload

Since RadRAT was developed to input data from a single patient at a time, we created batch mode programs written in Python to upload data for each synthetic patient (i.e., strata) and collated the results. These programs (available from the authors upon request) makes the process of analyzing multiple patients (whether real or synthetic) more efficient by three means: first by bypassing the website upload interface; second by bypassing the results interface; and third by automatically feeding in requests up to the load abilities of the RadRAT server. The batch tool takes inputs in the form of CSV data files (one per patient) and transforms them into the HTML form expected by the RadRAT backend and submits them directly to the server. The tool then watches for completion of the submission, loads the HTML response from the server, and transforms this output into a series of CSV files that are more easily processed than the HTML output. To manage this, the batch tool submits an initial, small batch of files for processing and automatically submits new requests whenever old requests are completed, keeping the RadRAT server full in terms of simultaneous working threads. We validated this batch upload approach by comparing it against standard, manual entry for a sample of synthetic patients and ensuring identical results.

Combined estimates across strata

To summarize results for some tables and figures, we combined estimates across strata. For combined point estimates, we calculated weighted sums of strata-specific rates. We calculated the combined uncertainty limits (UL) as the sum of the weighted strata-specific variances divided by the squared sum of the weighted strata-specific rates, where the weights are the proportions of exams contributed by each original stratum. Calculations were made on the natural logarithmic scale and converted back by exponentiation.

© 2025 Smith-Bindman R et al. *JAMA Internal Medicine.*

eMethods (continued)

CT categories used in cancer projections for adults and children

Body Region	CT Exam Category	Adults	Children
Abdomen and Pelvis	Low Dose	X	
	Routine Dose	X	X
	High Dose	X	X
Head	Low Dose	X	X
	Routine Dose	X	X
	High Dose	X	X
Chest	Low Dose	X	
	Routine Dose	X	
	Chest, All		X
Spine	Cervical Spine	X	X
	Thoracic or Lumbar	X	
	Thoracic + Lumbar Combined	X	
	Thoracic Spine		X
	Lumbar Spine		X
Head-neck	Routine Dose	X	
	High Dose	X	
Cardiac	Low Dose	X	
	Routine Dose	X	
	Cardiac, All		X
Cardiac/Chest	High Dose	X	
Combined Chest Abdomen and Pelvis*		X	
Whole body *			X
Extremity	Extremities	X	
	Upper Extremity Routine Dose		X
	Lower Extremity Routine Dose		X
	Lower Extremity High Dose		X

Footnote:
* Combined Chest Abdomen and Pelvis in adults and Whole Body in children are combined in the tables as Full Body.

© 2025 Smith-Bindman R et al. *JAMA Internal Medicine.*

eTable 1. Estimated number of computed tomography examinations performed in the U.S. in 2023, by age, sex, and CT category.

			Abdomen			Head			Chest		Spine	
**Sex**	**Age**	**All Exams**	**Low Dose**	**Routine Dose**	**High Dose**	**Low Dose**	**Routine Dose**	**High Dose**	**Low Dose**	**Routine Dose**	**Cervical Spine**	**Thoracic/ Lumbar Spine[a]**
All	All	93000000	1343600	26744300	2133500	3461400	19597500	1056900	193800	19035100	5694200	2041000
Child	All	3069000	NA	682500	7400	364900	1234900	1100	NA	275200	231900	58100
Adult	All	89931000	1343600	26061800	2126200	3096500	18362600	1055900	193800	18759900	5462300	1982900
Female	<1	97000	NA	4000	0	5000	67200	0	NA	13100	3100	0
Female	1-4	198500	NA	26300	0	20000	108400	0	NA	21500	16900	0
Female	5-9	240800	NA	56900	0	35900	91500	0	NA	21800	19000	2600
Female	10-14	386400	NA	103800	790	48000	129100	0	NA	30200	27300	13300
Female	15-17	483600	NA	164100	2000	46900	146500	0	NA	42800	38700	14200
Female	18-29	3663600	96300	1565100	34900	202300	688700	21700	0	438200	290200	47000
Female	30-39	4308900	119200	1804700	56600	191400	804200	39700	0	661000	266100	48200
Female	40-49	5434600	121600	2097300	95100	234800	958300	57800	0	909000	329900	87900
Female	50-59	7712500	115600	2543900	162500	264900	1349600	87900	22900	1606100	413000	173400
Female	60-69	9677300	119200	2635400	249200	278100	1740900	99900	45700	2475300	505700	262500
Female	70-79	9089800	72200	2265800	204700	204700	1851700	115600	34900	2301900	550200	281700
Female	80-89	5553800	40900	1384500	87900	125200	1514600	118000	0	1005300	354000	163700
Female	90-99	1702400	0	416600	22900	42100	555000	18100	0	219100	137200	37300
Male	<1	119800	NA	6300	0	7600	79400	0	NA	17400	2200	0
Male	1-4	249800	NA	31800	790	23900	143600	0	NA	21900	20600	0
Male	5-9	330300	NA	75300	860	53300	135500	0	NA	23300	24400	3900
Male	10-14	463800	NA	97800	790	68600	171600	0	NA	36100	34200	9700
Male	15-17	499000	NA	116400	2200	55700	162100	1100	NA	47000	45500	14500
Male	18-29	3516700	72200	1043800	30100	251600	831900	30100	0	343100	323900	60200
Male	30-39	3765900	86700	1218400	50600	214300	706700	36100	0	528500	279300	91500
Male	40-49	4556900	108400	1433900	90300	208300	892100	51800	0	753700	299800	77100
Male	50-59	7223700	121600	1964800	209500	264900	1380900	74600	18100	1594000	419000	136000
Male	60-69	9844600	139700	2492200	343100	275700	1926300	118000	40900	2562000	502000	202300
Male	70-79	8433600	87900	2009400	341900	208300	1662600	136000	31300	2209200	435800	196200
Male	80-89	4459400	42100	982400	146900	98700	1136500	50600	0	993300	275700	93900
Male	90-99	987200	0	203500	0	31300	362400	0	0	160100	80700	24100

© 2025 Smith-Bindman R et al. *JAMA Internal Medicine.*

eTable 1 (continued: number of exams within additional CT categories)

			Head and Neck Combined		Cardiac				
Sex	Age	All Exams	Routine Dose	High Dose	Low Dose	Routine Dose	Chest/ Cardiac High Dose	Full Body[b]	All Extremities[c]
All	All	93000000	2688400	138500	806600	267200	746400	4607000	2444500
Child	All	3069000	NA	NA	NA	26400	NA	57300	129300
Adult	All	89931000	2688400	138500	806600	240800	746400	4549700	2315200
Female	<1	97000	NA	NA	NA	4500	NA	0	0
Female	1-4	198500	NA	NA	NA	1400	NA	4100	0
Female	5-9	240800	NA	NA	NA	1800	NA	3800	7400
Female	10-14	386400	NA	NA	NA	2200	NA	4500	27200
Female	15-17	483600	NA	NA	NA	1800	NA	9900	16700
Female	18-29	3663600	103500	0	0	0	0	97500	78300
Female	30-39	4308900	86700	0	0	0	18100	121600	91500
Female	40-49	5434600	121600	0	66200	19300	24100	204700	107200
Female	50-59	7712500	187800	19300	138500	20500	28900	385300	192600
Female	60-69	9677300	190200	20500	133600	28900	55400	552600	284100
Female	70-79	9089800	272100	21700	47000	27700	97500	506900	233600
Female	80-89	5553800	304600	0	0	20500	63800	282900	87900
Female	90-99	1702400	166100	0	0	0	0	54200	33700
Male	<1	119800	NA	NA	NA	5900	NA	1100	0
Male	1-4	249800	NA	NA	NA	1900	NA	5300	0
Male	5-9	330300	NA	NA	NA	1600	NA	5500	6700
Male	10-14	463800	NA	NA	NA	3000	NA	7200	34900
Male	15-17	499000	NA	NA	NA	2300	NA	15800	36500
Male	18-29	3516700	158900	0	0	16900	15700	146900	191400
Male	30-39	3765900	119200	0	27700	14400	21700	197400	173400
Male	40-49	4556900	110800	0	79500	0	28900	250400	172200
Male	50-59	7223700	175800	18100	148100	21700	63800	394900	217900
Male	60-69	9844600	184200	26500	119200	33700	116800	567100	195000
Male	70-79	8433600	228700	16900	47000	37300	120400	492400	172200
Male	80-89	4459400	203500	15700	0	0	77100	258800	84300
Male	90-99	987200	74600	0	0	0	14400	36100	0

eTable 1 footnotes:
Row values may not sum to the "All Exams" column due to rounding.
[a] Thoracic and/or Lumbar Spine (one or both).
[b] Full Body includes Whole Body exams in children and combined Chest-Abdomen-Pelvis exams in adults.
[c] Extremity includes all upper and lower extremity exams.
NA = not applicable, meaning this category does not exist in this age group.

© 2025 Smith-Bindman R et al. *JAMA Internal Medicine.*

eTable 2. Sample computed tomography organ doses in mGy (mean, standard deviation) by CT category and sex, for exposures at age <1, 5-9 years, and 50-59 years. CT categories that were not represented in children are not shown.

Sex, Age	Organ	Abdomen Routine Dose	Head		Chest Routine Dose[a]	Cervical Spine	Cardiac Routine Dose[b]	Whole Body[c]
			Low Dose	Routine Dose				
Female	Bladder	20.5 (16.5)	0.1 (0.1)	0.2 (0.2)	2.1 (4.8)	7.4 (13.7)	1.0 (0.9)	-
< 1	Bone Marrow	4.5 (4.5)	11.7 (8.6)	26.2(16.4)	8.2 (7.5)	11.9 (7.0)	6.6 (4.5)	-
	Brain/CNS	0.4 (2.8)	39.6(25.4)	60.1(36.0)	4.3 (4.6)	12.4 (9.4)	2.0 (1.7)	-
	Breast	3.6 (9.1)	1.1 (1.4)	2.9 (2.6)	13.8(14.6)	17.8 (11.8)	12.3 (8.3)	-
	Colon	18.5 (17.5)	0.2 (0.3)	0.6 (0.5)	5.2 (6.6)	8.0 (14.4)	6.6 (5.5)	-
	Esophagus	1.9 (3.6)	6.5 (10.1)	23.9(18.0)	15.6(15.6)	19.3 (11.7)	13.6 (9.0)	-
	Gallbladder	13.5 (15.6)	0.5 (0.6)	1.3 (1.1)	9.9 (9.2)	8.9 (13.8)	13.5 (9.1)	-
	Kidney	13.4 (15.3)	0.4 (0.4)	1.0 (0.8)	10.3 (9.4)	9.2 (13.9)	13.5 (9.1)	-
	Liver	9.5 (12.1)	0.9 (1.3)	2.7 (2.4)	12.6 (11.8)	11.3 (12.8)	14.0 (9.3)	-
	Lung	3.1 (5.8)	5.4 (9.4)	15.5 (16.4)	15.6 (15.4)	19.9 (12.6)	14.2 (9.4)	-
	Oral Cavity[d]	0.5 (3.0)	20.4(21.8)	63.7(37.7)	13.8(13.7)	16.9 (12.1)	10.2 (6.9)	-
	Ovary	18.5 (15.4)	0.1 (0.1)	0.3 (0.3)	2.5 (5.0)	7.2 (13.3)	1.3 (1.2)	-
	Pancreas	14.3 (16.2)	0.5 (0.6)	1.3 (1.0)	9.3 (8.9)	8.8 (14.1)	13.4 (9.0)	-
	Rectum	18.2 (15.2)	0.1 (0.1)	0.3 (0.2)	2.1 (4.5)	6.6 (12.3)	1.0 (0.9)	-
	Stomach	10.6 (13.0)	0.6 (0.8)	1.8 (1.5)	12.1(10.9)	11.2 (13.2)	14.2 (9.4)	-
	Thyroid	1.0 (3.7)	18.3(24.5)	74.4(45.4)	19.8(19.9)	23.4 (15.7)	15.8 (10.5)	-
	Uterus	18.2 (15.2)	0.1 (0.1)	0.3 (0.2)	2.1 (4.5)	6.6 (12.3)	1.0 (0.9)	-
	Other	6.5 (6.5)	8.4 (8.2)	23.8(15.4)	11.3(10.2)	13.6 (9.4)	10.2 (6.9)	-
Male	Bladder	25.4 (21.0)	0.1 (0.1)	0.3 (0.4)	2.2 (7.1)	6.8 (15.6)	1.1 (0.7)	16.7 (8.9)
< 1	Bone Marrow	6.4 (5.1)	13.8(11.2)	26.7(16.7)	10.0(10.2)	18.7 (17.7)	6.4 (4.2)	11.2 (6.8)
	Brain/CNS	0.6 (4.4)	39.9 (26.4)	60.0 (36.5)	6.2 (7.9)	17.9 (19.8)	1.6 (3.0)	4.2 (11.5)
	Colon	24.1 (19.9)	0.3 (0.3)	0.7 (0.6)	5.5 (10.5)	8.7 (16.8)	6.7 (4.3)	21.0 (11.9)
	Esophagus	2.3 (3.6)	10.2 (12.7)	25.1 (18.2)	17.8 (18.0)	31.6 (31.1)	12.8 (8.5)	14.0 (7.8)
	Gallbladder	16.4 (15.8)	0.7 (0.7)	1.4 (1.1)	11.4 (14.6)	12.4 (17.8)	12.8 (8.1)	22.5 (15.8)
	Kidney	17.4 (16.3)	0.5 (0.5)	1.1 (0.8)	10.9 (14.1)	12.2 (18.3)	12.6 (8.0)	22.9 (15.9)
	Liver	11.9 (12.0)	1.3 (1.5)	2.8 (2.4)	13.7 (15.9)	18.9 (21.5)	13.1 (8.5)	21.9 (14.7)
	Lung	3.5 (4.8)	7.9 (10.6)	16.8 (16.8)	17.7 (18.2)	32.6 (33.1)	13.4 (9.0)	17.6 (9.0)
	Oral Cavity[d]	0.9 (4.9)	28.9 (29.6)	64.1 (38.4)	17.3 (19.0)	26.6 (28.2)	9.2 (6.2)	7.0 (13.1)
	Pancreas	18.9 (18.2)	0.6 (0.7)	1.3 (1.1)	10.2 (13.9)	11.1 (17.5)	12.5 (8.0)	22.9 (15.7)
	Prostate	24.5 (21.4)	0.1 (0.1)	0.2 (0.4)	1.4 (5.8)	6.0 (13.7)	0.5 (0.4)	13.7 (8.6)
	Rectum	24.5 (21.4)	0.1 (0.1)	0.2 (0.4)	1.4 (5.8)	6.0 (13.7)	0.5 (0.4)	13.7 (8.6)
	Stomach	13.8 (13.3)	0.9 (1.0)	1.9 (1.5)	13.1 (15.4)	16.5 (19.6)	13.2 (8.5)	22.4 (15.1)
	Thyroid	1.6 (6.1)	28.7 (34.0)	75.2 (46.4)	21.8 (22.1)	40.6 (41.5)	15.2 (10.6)	14.7 (14.7)
	Other	10.0 (7.8)	10.3 (9.9)	22.4 (14.3)	11.7 (12.2)	20.2 (19.8)	8.8 (5.6)	14.9 (8.3)

© 2025 Smith-Bindman R et al. *JAMA Internal Medicine.*

eTable2 (continued: organ doses (in mGy) for children aged 5-9 years)

Sex, Age	Organ	Abdomen Routine Dose	Head Low Dose	Head Routine Dose	Chest Routine Dose[a]	Spine Cervical Spine	Spine Thoracic Spine	Spine Lumbar Spine	Cardiac Routine Dose[b]	Whole Body[c]	Extremity Upper, Routine	Extremity Lower, Routine
Female 5-9	Bladder	12.7 (8.8)	0.0 (0.1)	0.0 (0.8)	2.9 (7.9)	2.5 (7.6)	13.1 (23.3)	15.3 (13.4)	1.3 (1.0)	12.2 (4.8)	0.2 (0.2)	0.6 (3.3)
	Bone Marrow	5.0 (3.4)	6.3 (5.5)	13.6 (8.7)	5.5 (6.0)	9.2 (6.2)	5.2 (7.6)	7.3 (8.2)	14.6 (16.8)	7.8 (4.1)	1.1 (7.0)	0.5 (1.8)
	Brain/CNS	0.9 (4.3)	21.8 (17.0)	45.7 (24.1)	3.7 (5.4)	16.3 (11.6)	1.0 (2.1)	2.7 (8.9)	2.2 (2.0)	2.7 (9.0)	2.6 (18.4)	1.0 (6.9)
	Breast	7.0 (6.7)	0.4 (1.8)	0.6 (1.1)	10.9 (12.2)	17.6 (13.6)	4.5 (7.3)	7.2 (11.3)	41.6 (51.0)	13.7 (8.8)	0.0 (0.3)	0.4 (2.8)
	Colon	12.8 (8.7)	0.1 (0.6)	0.1 (0.8)	7.6 (10.9)	3.1 (7.9)	13.8 (25.5)	16.2 (14.5)	13.0 (10.8)	15.9 (8.2)	0.0 (0.0)	0.5 (3.2)
	Esophagus	5.1 (4.9)	2.4 (5.7)	4.1 (5.9)	12.7 (13.2)	20.3 (15.6)	5.1 (8.4)	8.4 (13.5)	44.6 (53.6)	13.0 (8.2)	0.9 (6.2)	0.4 (2.8)
	Gallbladder	10.9 (7.7)	0.1 (0.7)	0.2 (0.7)	11.1 (12.9)	5.6 (7.7)	9.3 (14.8)	12.1 (13.5)	41.7 (51.3)	16.4 (8.9)	0.0 (0.1)	0.4 (3.2)
	Kidney	11.8 (8.0)	0.1 (0.7)	0.2 (0.7)	10.8 (12.7)	4.5 (7.7)	11.6 (20.9)	14.0 (14.7)	33.0 (35.5)	16.9 (9.2)	0.0 (0.1)	0.4 (3.3)
	Liver	10.1 (7.5)	0.2 (0.8)	0.3 (0.8)	11.9 (13.4)	9.8 (8.7)	7.6 (11.1)	10.6 (12.9)	45.5 (55.9)	16.4 (8.8)	0.0 (0.2)	0.4 (3.3)
	Lung	5.7 (5.9)	1.3 (4.0)	1.8 (2.2)	13.0 (14.2)	21.7 (16.6)	5.3 (8.5)	8.3 (13.3)	48.4 (60.2)	13.8 (8.8)	0.2 (1.5)	0.5 (3.1)
	Oral Cavity[d]	1.2 (4.3)	11.5 (16.3)	41.1 (28.7)	11.1 (9.8)	21.9 (16.4)	3.4 (8.5)	5.2 (10.7)	10.6 (8.5)	6.3 (10.8)	2.8 (20.3)	0.3 (1.3)
	Ovary	11.4 (7.9)	0.0 (0.3)	0.0 (0.7)	3.4 (8.0)	2.4 (7.4)	11.6 (20.9)	14.2 (12.0)	1.9 (1.5)	11.9 (5.0)	0.1 (0.1)	0.5 (3.1)
	Pancreas	11.4 (7.9)	0.1 (0.7)	0.2 (0.7)	10.9 (12.7)	4.9 (7.7)	11.1 (20.4)	13.1 (14.5)	38.2 (45.6)	16.7 (8.9)	0.0 (0.1)	0.4 (3.3)
	Rectum	10.9 (7.6)	0.0 (0.2)	0.0 (0.7)	3.0 (7.4)	2.2 (6.7)	11.1 (19.3)	13.5 (11.7)	1.6 (1.3)	11.2 (4.6)	0.1 (0.1)	0.5 (3.0)
	Stomach	10.8 (7.8)	0.2 (0.7)	0.3 (0.8)	11.9 (13.6)	8.1 (8.4)	9.2 (15.2)	11.8 (13.8)	45.3 (55.6)	16.9 (9.1)	0.0 (0.2)	0.4 (3.4)
	Thyroid	2.6 (6.1)	7.8 (18.7)	11.2 (20.7)	17.3 (16.2)	31.6 (27.0)	5.8 (12.2)	11.0 (19.1)	48.7 (57.2)	14.0 (14.2)	3.4 (24.3)	0.6 (3.2)
	Uterus	10.9 (7.6)	0.0 (0.2)	0.0 (0.7)	3.0 (7.4)	2.2 (6.7)	11.1 (19.3)	13.5 (11.7)	1.6 (1.3)	11.2 (4.6)	0.1 (0.1)	0.5 (3.0)
	Other	6.3 (4.4)	3.7 (4.9)	10.2 (7.3)	9.4 (9.7)	13.1 (9.2)	6.1 (8.7)	8.9 (10.6)	25.7 (28.4)	11.3 (5.6)	1.0 (6.1)	0.6 (2.2)
Male 5-9	Bladder	13.0 (9.4)	0.0 (0.5)	0.1 (1.1)	3.5 (8.2)	2.7 (8.3)	13.5 (11.0)	15.5 (18.6)	1.0 (0.6)	12.8 (5.4)	0.2 (0.2)	0.3 (0.4)
	Bone Marrow	5.2 (3.9)	7.1 (5.8)	14.6 (10.0)	6.9 (7.8)	9.4 (6.7)	5.6 (5.1)	7.6 (11.3)	8.8 (7.3)	9.2 (4.8)	0.6 (2.2)	0.2 (0.9)
	Brain/CNS	0.7 (3.8)	24.9 (18.0)	48.0 (27.3)	4.9 (10.9)	16.6 (13.3)	0.2 (0.4)	4.0 (11.8)	1.7 (1.3)	2.9 (9.0)	2.3 (10.7)	0.3 (1.9)
	Colon	13.4 (9.7)	0.1 (0.8)	0.1 (1.2)	9.2 (11.5)	3.4 (8.9)	15.9 (14.3)	16.7 (20.7)	9.1 (6.0)	19.7 (10.8)	0.1 (0.1)	0.1 (0.1)
	Esophagus	5.4 (5.6)	2.5 (5.5)	5.3 (8.2)	16.0 (18.2)	20.2 (16.5)	5.4 (8.5)	9.7 (20.4)	25.8 (22.8)	15.2 (7.4)	0.1 (0.4)	0.2 (1.2)
	Gallbladder	11.2 (8.8)	0.1 (0.8)	0.2 (1.2)	13.3 (13.2)	6.0 (8.8)	12.7 (13.2)	13.4 (20.2)	24.4 (22.3)	21.0 (13.2)	0.0 (0.0)	0.0 (0.0)
	Kidney	12.2 (9.2)	0.1 (0.9)	0.3 (1.2)	12.9 (13.4)	4.9 (8.7)	14.3 (14.0)	14.7 (20.6)	20.0 (15.9)	21.4 (13.2)	0.0 (0.0)	0.0 (0.0)
	Liver	10.6 (8.8)	0.2 (0.9)	0.4 (1.4)	14.3 (14.3)	10.2 (10.1)	11.2 (13.4)	12.4 (19.9)	26.3 (24.7)	20.9 (13.2)	0.0 (0.0)	0.0 (0.1)
	Lung	6.1 (6.8)	1.3 (3.3)	2.4 (4.4)	16.4 (19.1)	21.4 (17.4)	5.8 (9.3)	9.5 (20.4)	27.9 (26.8)	16.0 (8.1)	0.0 (0.2)	0.1 (0.8)
	Oral Cavity[d]	1.1 (3.6)	13.7 (19.5)	45.4 (31.3)	14.0 (16.6)	22.6 (17.6)	1.3 (4.5)	7.1 (18.9)	9.3 (8.1)	5.1 (8.8)	1.4 (8.6)	0.3 (2.1)
	Pancreas	11.8 (9.1)	0.1 (0.8)	0.2 (1.2)	13.1 (13.4)	5.2 (8.7)	13.8 (13.9)	14.5 (20.6)	22.6 (20.0)	21.5 (13.5)	0.0 (0.0)	0.0 (0.0)
	Prostate	12.4 (9.2)	0.0 (0.4)	0.1 (1.0)	2.5 (7.1)	2.5 (7.8)	9.5 (7.8)	12.7 (17.0)	0.4 (0.3)	10.2 (5.0)	0.4 (0.3)	0.4 (0.5)
	Rectum	12.4 (9.2)	0.0 (0.4)	0.1 (1.0)	2.5 (7.1)	2.5 (7.8)	9.5 (7.8)	12.7 (17.0)	0.4 (0.3)	10.2 (5.0)	0.4 (0.3)	0.4 (0.5)
	Stomach	11.3 (9.1)	0.2 (0.9)	0.4 (1.3)	14.4 (14.5)	8.6 (9.6)	12.4 (14.0)	13.5 (20.6)	26.3 (24.7)	21.7 (13.8)	0.0 (0.0)	0.0 (0.0)
	Thyroid	2.6 (6.6)	8.0 (18.8)	14.3 (26.8)	21.9 (27.0)	31.4 (28.1)	3.7 (9.8)	12.3 (30.8)	29.8 (22.5)	15.2 (8.0)	0.2 (1.3)	0.5 (3.5)
	Other	7.2 (5.4)	3.9 (4.8)	10.5 (7.9)	10.8 (11.4)	12.5 (9.3)	6.7 (7.0)	9.7 (16.0)	14.5 (11.4)	12.8 (6.1)	0.6 (1.8)	0.4 (0.8)

© 2025 Smith-Bindman R et al. *JAMA Internal Medicine.*

eTable2 (continued: organ doses (in mGy) for adults aged 50-59 years, Abdomen and Pelvis, Head, and Chest)

Sex, Age	Organ	Abdomen and Pelvis			Head			Chest	
		Low Dose	Routine Dose	High Dose	Low Dose	Routine Dose	High Dose	Low Dose	Routine Dose[a]
Female	Bladder	15.5 (10.1)	20.4 (13.2)	49.7 (30.5)	0.0 (0.0)	0.2 (1.9)	0.0 (0.0)	0.2 (0.5)	4.0 (8.0)
50-59	Bone Marrow	9.3 (5.9)	11.8 (6.8)	27.8 (18.5)	1.4 (2.5)	3.6 (3.2)	5.9 (5.4)	2.9 (4.8)	11.4 (8.0)
	Brain/CNS	0.1 (0.1)	0.5 (4.4)	2.9 (8.4)	26.3 (24.5)	58.5 (33.7)	69.6 (43.6)	0.7 (1.0)	1.9 (3.2)
	Breast	14.0 (11.4)	16.0 (11.5)	32.4 (32.0)	0.1 (0.2)	0.5 (2.5)	0.5 (0.5)	6.0 (9.3)	25.2 (19.3)
	Colon	20.4 (12.6)	25.4 (15.2)	58.6 (35.5)	0.0 (0.0)	0.2 (2.8)	0.0 (0.0)	3.2 (7.1)	14.1 (14.4)
	Esophagus	8.1 (6.9)	10.3 (8.3)	23.3 (25.3)	1.0 (5.3)	2.2 (5.0)	8.2 (10.9)	6.8 (11.0)	24.2 (15.9)
	Gallbladder	17.6 (11.6)	20.4 (11.1)	39.1 (32.2)	0.0 (0.2)	0.3 (2.6)	0.3 (0.3)	6.0 (9.8)	23.0 (16.4)
	Kidney	20.8 (13.5)	24.4 (13.2)	48.3 (35.8)	0.0 (0.1)	0.3 (3.0)	0.1 (0.1)	6.0 (10.6)	22.5 (17.0)
	Liver	18.6 (12.7)	21.6 (11.9)	41.6 (34.4)	0.1 (0.2)	0.4 (2.8)	0.4 (0.5)	6.6 (10.6)	25.9 (18.6)
	Lung	9.8 (9.1)	11.8 (10.2)	26.5 (30.0)	0.7 (3.2)	1.5 (3.6)	5.2 (6.9)	6.8 (10.4)	26.1 (17.9)
	Oral Cavity[d]	0.4 (0.5)	1.7 (7.6)	7.0 (14.8)	14.6 (20.0)	56.0 (36.5)	63.2 (50.2)	11.2 (18.9)	26.9 (24.2)
	Ovary	14.3 (9.4)	19.1 (13.0)	49.6 (29.1)	0.0 (0.0)	0.2 (1.9)	0.0 (0.0)	0.3 (0.7)	4.5 (8.4)
	Pancreas	19.2 (12.5)	22.4 (12.0)	43.7 (33.8)	0.0 (0.1)	0.3 (2.7)	0.2 (0.2)	5.9 (9.9)	22.3 (16.3)
	Rectum	12.8 (8.3)	17.1 (11.4)	43.4 (25.9)	0.0 (0.0)	0.1 (1.6)	0.0 (0.0)	0.3 (0.6)	3.8 (7.2)
	Stomach	20.3 (13.6)	23.4 (12.7)	44.5 (35.7)	0.0 (0.2)	0.4 (3.0)	0.3 (0.4)	6.8 (11.0)	26.5 (18.9)
	Thyroid	1.6 (2.1)	4.9 (12.1)	15.4 (27.5)	5.1 (26.4)	10.8 (25.5)	39.8 (52.0)	13.2 (22.0)	37.5 (25.5)
	Uterus	12.8 (8.3)	17.1 (11.4)	43.4 (25.9)	0.0 (0.0)	0.1 (1.6)	0.0 (0.0)	0.3 (0.6)	3.8 (7.2)
	Other	10.2 (6.7)	12.6 (7.4)	27.7 (20.8)	3.5 (6.8)	11.0 (9.5)	16.6 (15.4)	5.6 (9.2)	19.2 (12.0)
Male	Bladder	19.5 (11.8)	24.0 (16.3)	55.0 (31.6)	0.0 (0.0)	0.1 (1.2)	0.0 (0.0)	0.1 (0.0)	3.5 (8.6)
50-59	Bone Marrow	10.0 (6.4)	12.2 (7.4)	29.0 (19.9)	1.4 (1.1)	3.5 (2.7)	5.8 (5.1)	1.6 (0.6)	10.8 (8.2)
	Brain/CNS	0.0 (0.1)	0.8 (6.0)	6.1 (14.2)	22.6 (13.6)	48.5 (26.4)	64.3 (41.3)	0.4 (0.2)	2.1 (4.4)
	Colon	22.5 (13.7)	27.1 (17.6)	63.5 (38.4)	0.0 (0.0)	0.1 (1.5)	0.0 (0.0)	1.0 (0.8)	13.1 (15.7)
	Esophagus	9.9 (7.6)	11.4 (9.1)	27.2 (30.2)	0.3 (0.3)	1.2 (2.7)	5.6 (7.1)	4.0 (1.7)	24.4 (17.9)
	Gallbladder	18.9 (11.5)	21.5 (12.8)	48.0 (36.5)	0.0 (0.0)	0.2 (1.6)	0.1 (0.1)	2.5 (1.2)	20.7 (18.6)
	Kidney	21.7 (13.6)	25.1 (15.6)	56.8 (38.2)	0.0 (0.0)	0.2 (1.6)	0.1 (0.1)	1.8 (1.3)	18.9 (19.0)
	Liver	20.3 (12.6)	21.7 (12.4)	46.9 (38.7)	0.0 (0.0)	0.2 (1.8)	0.2 (0.3)	3.7 (1.5)	25.6 (20.6)
	Lung	11.8 (10.3)	13.1 (11.0)	30.0 (34.3)	0.2 (0.2)	0.9 (2.0)	3.1 (3.8)	4.5 (1.9)	27.2 (20.1)
	Oral Cavity[d]	0.2 (0.3)	1.8 (9.0)	8.2 (17.7)	14.5 (18.1)	37.6 (38.9)	55.4 (57.1)	4.8 (1.9)	21.4 (16.2)
	Pancreas	20.3 (12.6)	23.3 (14.2)	52.4 (37.1)	0.0 (0.0)	0.1 (1.5)	0.1 (0.1)	2.0 (1.3)	19.3 (18.4)
	Prostate	20.1 (12.3)	22.9 (14.2)	46.0 (31.1)	0.0 (0.0)	0.1 (1.2)	0.0 (0.0)	0.0 (0.0)	2.4 (7.1)
	Rectum	20.1 (12.3)	22.9 (14.2)	46.0 (31.1)	0.0 (0.0)	0.1 (1.2)	0.0 (0.0)	0.0 (0.0)	2.4 (7.1)
	Stomach	21.6 (13.5)	23.1 (13.2)	50.0 (40.4)	0.0 (0.0)	0.2 (1.8)	0.2 (0.2)	3.7 (1.5)	26.1 (21.2)
	Thyroid	1.3 (2.0)	5.8 (16.3)	21.3 (38.1)	2.2 (3.0)	8.4 (20.7)	45.1 (57.7)	9.3 (4.0)	44.9 (31.4)
	Other	13.1 (8.5)	15.3 (8.8)	34.5 (25.8)	3.0 (3.0)	8.2 (6.6)	13.2 (12.6)	3.1 (1.3)	18.6 (12.6)

© 2025 Smith-Bindman R et al. *JAMA Internal Medicine.*

eTable2 (continued: organ doses (in mGy) for adults aged 50-59 years, remaining CT categories)

Sex, Age	Organ	Spine			Head and Neck Combined		Cardiac		Chest or Cardiac High Dose	Combined Chest, Abdomen & Pelvis	Extremity
		Cervical Spine	Thoracic or Lumbar	Thoracic & Lumbar	Routine Dose	High Dose	Low Dose	Routine Dose			
Female	Bladder	3.8 (12.6)	28.5 (16.6)	35.2 (29.4)	2.3 (7.3)	0.1 (0.0)	0.1 (0.1)	3.9 (12.2)	10.1 (16.6)	19.1 (14.5)	1.6 (2.4)
50-59	Bone Marrow	12.9 (10.6)	19.0 (11.7)	30.2 (22.7)	16.3 (14.9)	21.5 (11.6)	3.2 (4.8)	40.3 (35.3)	22.5 (22.0)	17.9 (10.8)	0.5 (1.3)
	Brain/CNS	30.2 (25.8)	0.6 (4.3)	8.6 (19.2)	34.4 (34.5)	21.9 (32.7)	0.2 (0.3)	1.6 (1.4)	7.3 (10.4)	3.2 (10.6)	0.1 (1.8)
	Breast	21.2 (23.0)	13.2 (18.2)	34.2 (33.5)	14.0 (15.0)	15.0 (10.4)	8.9 (15.8)	113.0 (93.9)	53.3 (55.0)	30.7 (18.3)	0.2 (1.9)
	Colon	5.1 (15.6)	50.8 (28.9)	59.5 (37.4)	3.7 (9.9)	0.5 (0.3)	1.3 (1.5)	19.0 (29.1)	28.7 (28.8)	33.8 (21.8)	0.5 (2.3)
	Esophagus	31.8 (25.1)	11.1 (13.0)	35.7 (36.0)	41.8 (46.2)	61.9 (37.2)	8.9 (13.6)	113.1 (103.3)	46.5 (44.8)	24.6 (15.3)	0.2 (1.6)
	Gallbladder	7.4 (13.7)	37.6 (29.1)	52.4 (32.2)	6.4 (9.3)	4.3 (2.7)	7.8 (14.0)	107.6 (100.2)	44.2 (42.4)	31.4 (21.2)	0.3 (2.1)
	Kidney	6.0 (14.8)	49.4 (30.2)	59.8 (35.9)	4.8 (10.1)	1.6 (1.0)	4.8 (6.6)	65.6 (78.3)	41.3 (36.5)	35.5 (23.7)	0.3 (2.4)
	Liver	10.5 (16.0)	34.6 (27.6)	52.5 (33.7)	8.5 (10.2)	7.0 (4.4)	8.8 (15.2)	117.1 (105.5)	52.3 (52.5)	34.6 (22.5)	0.3 (2.3)
	Lung	34.1 (28.9)	10.3 (15.2)	35.8 (36.8)	41.0 (44.9)	59.0 (36.4)	10.7 (17.1)	127.8 (110.0)	52.9 (53.6)	27.6 (16.7)	0.2 (1.8)
	Oral Cavity[d]	46.6 (33.2)	1.4 (7.7)	23.2 (38.9)	57.2 (35.4)	50.1 (27.1)	2.2 (3.5)	10.1 (9.1)	35.9 (19.1)	11.8 (16.7)	0.2 (2.7)
	Ovary	3.8 (12.6)	35.7 (21.2)	42.6 (28.6)	2.5 (7.8)	0.1 (0.0)	0.1 (0.1)	3.9 (10.1)	11.8 (18.9)	19.8 (14.5)	0.9 (1.8)
	Pancreas	6.2 (14.0)	45.4 (29.7)	57.3 (34.5)	5.4 (9.7)	2.7 (1.7)	5.8 (8.0)	76.3 (87.9)	42.0 (38.3)	33.2 (22.2)	0.3 (2.3)
	Rectum	3.3 (11.0)	29.8 (17.9)	36.0 (25.5)	2.1 (6.7)	0.1 (0.0)	0.1 (0.1)	3.5 (9.3)	9.5 (15.1)	17.2 (12.6)	1.1 (1.8)
	Stomach	9.0 (15.6)	39.7 (29.6)	56.3 (34.4)	7.5 (10.2)	5.5 (3.5)	8.6 (14.0)	111.4 (104.1)	53.2 (52.7)	36.3 (23.6)	0.3 (2.4)
	Thyroid	68.8 (52.1)	2.9 (11.9)	44.4 (63.7)	114.2 (134.9)	182.3 (106.7)	8.3 (6.9)	108.3 (147.5)	57.5 (49.4)	27.8 (27.2)	0.2 (1.9)
	Uterus	3.3 (11.0)	29.8 (17.9)	36.0 (25.5)	2.1 (6.7)	0.1 (0.0)	0.1 (0.1)	3.5 (9.3)	9.5 (15.1)	17.2 (12.6)	1.1 (1.8)
	Other	23.4 (16.1)	19.0 (12.7)	35.7 (30.1)	33.5 (30.3)	45.7 (23.8)	5.1 (7.2)	61.7 (56.1)	35.0 (30.0)	22.5 (13.1)	0.8 (1.8)
Male	Bladder	11.0 (21.0)	35.2 (19.4)	-	3.7 (9.3)	0.0 (0.0)	0.1 (0.1)	1.3 (1.8)	10.0 (15.5)	22.2 (14.4)	0.4 (3.2)
50-59	Bone Marrow	14.3 (11.2)	20.4 (12.4)	-	14.2 (10.0)	13.6 (9.5)	2.9 (2.9)	33.0 (33.3)	23.0 (24.2)	18.5 (10.4)	0.5 (2.3)
	Brain/CNS	21.2 (21.0)	0.1 (0.3)	-	39.3 (33.3)	33.8 (25.4)	0.2 (0.2)	2.9 (7.0)	5.2 (8.2)	1.4 (5.5)	0.1 (1.3)
	Colon	13.4 (25.1)	54.8 (29.2)	-	5.0 (12.4)	0.2 (0.2)	0.8 (1.4)	18.1 (25.4)	30.1 (31.0)	39.6 (21.8)	0.3 (3.6)
	Esophagus	28.2 (22.2)	12.0 (15.2)	-	31.3 (29.0)	33.8 (27.8)	9.1 (8.8)	96.9 (98.2)	52.1 (60.9)	25.8 (15.7)	0.3 (3.3)
	Gallbladder	13.6 (23.3)	47.0 (30.2)	-	5.6 (11.4)	0.9 (0.8)	4.6 (6.5)	76.4 (97.2)	47.0 (50.4)	36.7 (21.5)	0.3 (3.4)
	Kidney	13.5 (24.0)	52.8 (29.7)	-	5.3 (12.1)	0.5 (0.4)	2.4 (3.6)	47.9 (63.8)	43.4 (47.0)	41.1 (23.1)	0.3 (3.4)
	Liver	14.5 (22.8)	40.6 (29.6)	-	7.0 (11.2)	2.5 (2.1)	7.4 (9.5)	98.7 (107.8)	59.0 (66.6)	38.4 (21.9)	0.3 (3.6)
	Lung	29.2 (24.6)	11.3 (17.2)	-	30.6 (29.1)	32.6 (27.3)	10.8 (11.5)	113.5 (111.9)	59.1 (69.0)	29.5 (17.2)	0.3 (3.6)
	Oral Cavity [d]	40.9 (33.0)	1.1 (7.9)	-	79.7 (67.6)	91.0 (64.2)	2.7 (4.2)	11.8 (19.8)	21.7 (14.9)	6.2 (11.5)	0.2 (2.3)
	Pancreas	13.1 (23.3)	50.9 (29.4)	-	5.2 (11.6)	0.6 (0.5)	2.5 (3.9)	50.7 (71.3)	43.6 (46.5)	38.5 (21.9)	0.3 (3.3)
	Prostate	9.3 (17.8)	12.7 (11.3)	-	2.9 (7.6)	0.0 (0.0)	0.0 (0.0)	0.4 (0.6)	5.1 (9.6)	16.6 (15.2)	0.7 (3.4)
	Rectum	9.3 (17.8)	12.7 (11.3)	-	2.9 (7.6)	0.0 (0.0)	0.0 (0.0)	0.4 (0.6)	5.1 (9.6)	16.6 (15.2)	0.7 (3.4)
	Stomach	14.9 (24.1)	45.4 (31.1)	-	6.7 (12.0)	1.7 (1.5)	6.9 (9.1)	92.9 (106.1)	59.5 (66.0)	40.4 (22.9)	0.3 (3.7)
	Thyroid	63.2 (51.6)	4.7 (20.0)	-	103.4 (114.9)	138.6 (111.6)	9.4 (7.0)	87.8 (105.3)	66.5 (64.6)	28.8 (32.2)	0.4 (4.0)
	Other	22.2 (14.6)	19.9 (13.4)	-	27.0 (20.1)	28.1 (19.7)	4.6 (4.5)	50.2 (50.1)	35.5 (34.4)	24.5 (13.6)	0.8 (2.9)

© 2025 Smith-Bindman R et al. *JAMA Internal Medicine.*

eTable2 (continued: footnotes)

eTable 2 footnotes:
Dashes (-) mean fewer than 12 exams occurred in this category in the UCSF CT registry; thus it was not included in calculations.
[a] Chest Routine Dose includes all chest exams in children.
[b] Cardiac Routine Dose includes all cardiac exams in children.
[c] Whole Body exams in children are combined with Chest-Abdomen-Pelvis exams in adults to create the category of Full Body as shown in other Tables.
[d] Oral Cavity includes Pharynx.

© 2025 Smith-Bindman R et al. *JAMA Internal Medicine.*

eTable 3. Projected number of future cancers (with uncertainty limits, UL) overall and by cancer type that could result from computed tomography exams performed in the U.S. in 2023, by body region, age group (adults vs children) and sex.

Body region	Age group - Sex	CT Exams	Total Projected Cancers (UL)	Cancer Type (UL)							
				Lung	Colon	Leukemia	Bladder	Stomach	Thyroid	Breast	Liver
All	All - All	93000000	102700 (96400, 109500)	22400 (20200, 25000)	8700 (7800, 9700)	7900 (6700, 9500)	7100 (6000, 8500)	7100 (5500, 9100)	7000 (5400, 9200)	5700 (5000, 6500)	4100 (3400, 5000)
	Child - All	3069000	9700 (8100, 11600)	990 (870, 1100)	330 (280, 390)	550 (380, 820)	250 (200, 320)	280 (200, 400)	3500 (2300, 5500)	630 (550, 730)	160 (130, 200)
	Adult - All	89931000	93000 (86900, 99600)	21400 (19200, 24000)	8400 (7500, 9400)	7400 (6100, 8900)	6900 (5700, 8200)	6800 (5200, 8800)	3500 (2700, 4600)	5100 (4400, 5900)	4000 (3200, 4900)
Abdomen and Pelvis	Adult - F	16312200	20200 (16900, 24200)	4100 (3100, 5500)	2200 (1600, 2900)	1400 (850, 2200)	2500 (1800, 3400)	1900 (1000, 3600)	190 (100, 370)	2400 (1800, 3100)	720 (420, 1300)
	Adult - M	13219300	17200 (14300, 20700)	1600 (1200, 2100)	2800 (2300, 3500)	1600 (1000, 2600)	2600 (1900, 3700)	1300 (690, 2400)	50 (20, 90)	NA	1000 (680, 1600)
	Child - F	357700	920 (710, 1200)	140 (90, 220)	80 (50, 120)	30 (14, 50)	90 (50, 140)	70 (30, 180)	60 (30, 100)	220 (160, 300)	20 (11, 60)
	Child - M	332100	700 (530, 940)	50 (30, 70)	120 (90, 170)	30 (19, 50)	100 (60, 150)	60 (20, 140)	11 (6, 20)	NA	40 (20, 80)
Head	Adult - F	11565100	3700 (2900, 4700)	570 (440, 720)	15 (11, 20)	300 (190, 490)	13 (9, 18)	20 (14, 40)	510 (290, 920)	70 (50, 80)	11 (7, 19)
	Adult - M	10949900	3700 (2900, 4700)	250 (200, 320)	18 (14, 20)	430 (280, 660)	12 (8, 17)	17 (9, 30)	150 (80, 250)	NA	17 (11, 30)
	Child - F	698500	3000 (1800, 5000)	170 (110, 260)	2 (2, 3)	160 (70, 370)	1 (1, 2)	6 (3, 16)	2000 (960, 4300)	60 (50, 80)	3 (1, 7)
	Child - M	902300	2100 (1600, 2800)	100 (70, 160)	4 (3, 6)	250 (130, 480)	2 (1, 3)	7 (3, 19)	520 (260, 1000)	NA	7 (4, 14)
Chest	Adult - F	9719400	12700 (10200, 15900)	5100 (3700, 7000)	570 (420, 790)	720 (370, 1400)	210 (140, 310)	1100 (510, 2200)	660 (340, 1300)	1400 (1100, 1800)	430 (220, 840)
	Adult - M	9234200	8800 (7100, 10800)	2100 (1500, 3000)	680 (510, 900)	880 (450, 1700)	200 (130, 310)	800 (360, 1800)	180 (90, 340)	NA	690 (390, 1200)
	Child - F	129500	780 (590, 1000)	140 (100, 200)	16 (11, 20)	15 (7, 30)	7 (4, 12)	30 (15, 70)	300 (170, 510)	160 (120, 210)	12 (6, 20)
	Child - M	145700	400 (320, 500)	70 (50, 100)	30 (20, 40)	20 (11, 40)	8 (5, 14)	30 (13, 70)	70 (40, 110)	NA	20 (14, 40)

© 2025 Smith-Bindman R et al. *JAMA Internal Medicine.*

eTable 3 (continued: from previous page with additional body regions)

Body region	Age group - Sex	CT Exams	Total Projected Cancers (UL)	Cancer Type (UL)							
				Lung	Colon	Leukemia	Bladder	Stomach	Thyroid	Breast	Liver
Spine	Adult - F	3947700	6700 (5600, 8200)	2400 (1800, 3200)	320 (250, 410)	390 (250, 600)	260 (190, 350)	300 (180, 500)	860 (390, 1900)	600 (470, 770)	120 (80, 190)
	Adult - M	3497500	4900 (4200, 5600)	800 (600, 1100)	570 (480, 690)	480 (320, 720)	400 (300, 540)	290 (170, 490)	180 (80, 380)	NA	230 (160, 330)
	Child - F	135100	800 (600, 1100)	150 (100, 200)	14 (11, 18)	15 (9, 30)	12 (9, 17)	18 (10, 30)	360 (210, 620)	130 (100, 160)	7 (4, 12)
	Child - M	155000	470 (380, 590)	80 (60, 120)	30 (20, 30)	30 (15, 40)	17 (11, 20)	20 (10, 40)	100 (50, 170)	NA	18 (11, 30)
Head and Neck Combined	Adult - F	1494100	2500 (2000, 3100)	1100 (830, 1500)	20 (16, 30)	140 (80, 240)	19 (13, 30)	40 (20, 80)	470 (230, 960)	130 (100, 170)	18 (10, 30)
	Adult - M	1332800	1600 (1300, 2000)	350 (260, 480)	50 (30, 60)	160 (90, 260)	30 (20, 50)	30 (15, 70)	130 (60, 300)	NA	30 (17, 50)
Cardiac	Adult - F	789800	1500 (1300, 1700)	760 (630, 910)	40 (30, 60)	90 (50, 160)	14 (10, 20)	140 (80, 240)	20 (14, 40)	120 (110, 140)	60 (40, 100)
	Adult - M	1004100	1800 (1500, 2000)	510 (420, 640)	100 (80, 120)	170 (100, 290)	30 (20, 50)	170 (100, 300)	30 (18, 50)	NA	160 (100, 230)
	Child - F	11800	120 (90, 160)	30 (19, 40)	2 (1, 3)	2 (1, 6)	0 (0, 0)	6 (3, 14)	40 (20, 70)	30 (20, 40)	2 (1, 5)
	Child - M	14600	50 (40, 70)	11 (7, 15)	3 (2, 4)	3 (1, 6)	0 (0, 1)	5 (2, 12)	8 (4, 15)	NA	4 (2, 7)
Full Body	Adult - F	2205600	3700 (3000, 4500)	1100 (820, 1600)	330 (240, 470)	250 (130, 500)	240 (160, 360)	340 (160, 700)	50 (30, 90)	370 (290, 470)	130 (70, 270)
	Adult - M	2344100	4000 (3300, 4800)	590 (420, 830)	640 (500, 830)	370 (200, 680)	340 (220, 520)	360 (170, 780)	30 (14, 50)	NA	300 (180, 510)
	Child - F	22400	150 (110, 200)	30 (18, 40)	8 (5, 13)	3 (2, 7)	7 (4, 11)	9 (3, 20)	30 (18, 60)	30 (30, 50)	3 (1, 8)
	Child - M	34900	170 (130, 220)	20 (15, 30)	20 (17, 30)	7 (4, 13)	12 (7, 20)	16 (6, 40)	12 (6, 20)	NA	12 (6, 20)
Extremity	Adult - F	1108800	30 (20, 40)	3 (2, 5)	2 (1, 3)	3 (2, 6)	9 (6, 14)	1 (0, 2)	0 (0, 0)	1 (0, 1)	0 (0, 1)
	Adult - M	1206400	40 (30, 50)	3 (2, 4)	3 (2, 4)	5 (3, 9)	4 (2, 6)	1 (0, 3)	0 (0, 0)	NA	1 (0, 2)
	Child - F	51300	5 (4, 8)	0 (0, 1)	0 (0, 0)	0 (0, 0)	0 (0, 0)	0 (0, 0)	3 (1, 5)	0 (0, 0)	0 (0, 0)
	Child - M	78000	3 (2, 4)	0 (0, 0)	0 (0, 0)	0 (0, 0)	0 (0, 0)	0 (0, 0)	0 (0, 0)	NA	0 (0, 0)

© 2025 Smith-Bindman R et al. *JAMA Internal Medicine.*

eTable 3 (continued: with additional cancer types)

Body region	Age group - Sex	Cancer Type (UL)									
		**Kidney**	**Pancreas**	**Oral Cavity / Pharynx**	**Brain / CNS**	**Esophagus**	**Prostate**	**Ovary**	**Rectum**	**Uterus**	**Other and ill-defined sites**
All	All - All	3000 (2300, 3900)	2800 (2300, 3500)	2800 (2300, 3400)	1600 (1300, 2000)	1500 (1300, 1800)	1500 (820, 2700)	890 (670, 1200)	560 (480, 660)	550 (400, 760)	17400 (15300, 19800)
	Child - All	130 (90, 180)	100 (80, 140)	450 (310, 650)	440 (320, 620)	110 (90, 150)	70 (30, 170)	40 (30, 70)	30 (20, 40)	30 (16, 50)	1600 (1200, 2000)
	Adult - All	2900 (2200, 3700)	2700 (2200, 3400)	2300 (1900, 2900)	1200 (910, 1500)	1400 (1200, 1700)	1400 (760, 2700)	850 (630, 1100)	540 (450, 630)	530 (380, 730)	15800 (13700, 18200)
Abdomen and Pelvis	Adult - F	670 (370, 1200)	760 (460, 1300)	30 (17, 40)	3 (2, 5)	110 (90, 140)	NA	620 (410, 930)	190 (140, 250)	390 (250, 600)	2100 (1300, 3500)
	Adult - M	850 (460, 1600)	630 (400, 1000)	30 (17, 60)	19 (12, 30)	250 (170, 380)	1100 (520, 2500)	NA	220 (160, 310)	NA	3100 (2100, 4400)
	Child - F	30 (12, 60)	30 (13, 60)	1 (1, 2)	0 (0, 0)	4 (3, 6)	NA	30 (17, 50)	9 (6, 15)	20 (10, 40)	90 (40, 190)
	Child - M	40 (17, 90)	30 (13, 50)	1 (1, 2)	1 (1, 2)	9 (5, 17)	50 (18, 160)	NA	13 (7, 20)	NA	150 (90, 240)
Head	Adult - F	6 (3, 10)	8 (5, 13)	750 (480, 1200)	220 (150, 310)	20 (20, 30)	NA	4 (2, 5)	1 (1, 2)	2 (2, 3)	1100 (720, 1800)
	Adult - M	8 (4, 15)	6 (4, 9)	590 (340, 1000)	710 (500, 1000)	60 (40, 80)	5 (4, 6)	NA	1 (1, 2)	NA	1400 (1000, 2000)
	Child - F	1 (1, 3)	1 (1, 3)	170 (100, 260)	70 (50, 110)	11 (5, 20)	NA	1 (0, 1)	0 (0, 0)	0 (0, 1)	330 (180, 580)
	Child - M	3 (1, 6)	2 (1, 4)	230 (120, 430)	350 (230, 530)	40 (20, 80)	1 (1, 1)	NA	0 (0, 0)	NA	600 (390, 940)
Chest	Adult - F	280 (150, 530)	360 (200, 650)	180 (90, 350)	4 (2, 6)	140 (100, 210)	NA	70 (40, 110)	18 (17, 20)	40 (30, 50)	1500 (850, 2600)
	Adult - M	320 (150, 700)	280 (150, 500)	170 (90, 350)	15 (9, 20)	320 (190, 520)	60 (40, 90)	NA	12 (10, 14)	NA	2100 (1300, 3300)
	Child - F	9 (4, 19)	10 (5, 19)	7 (4, 12)	1 (0, 1)	5 (3, 8)	NA	3 (2, 6)	1 (1, 1)	2 (1, 4)	60 (30, 110)
	Child - M	15 (7, 30)	10 (6, 19)	11 (6, 20)	4 (2, 6)	14 (8, 20)	3 (1, 8)	NA	1 (1, 1)	NA	90 (60, 150)

© 2025 Smith-Bindman R et al. *JAMA Internal Medicine.*

eTable 3 (continued: from previous page)

Body region	Age group - Sex	Cancer Type (UL)									
		Kidney	Pancreas	Oral Cavity / Pharynx	Brain / CNS	Esophagus	Prostate	Ovary	Rectum	Uterus	Other and ill-defined sites
Spine	Adult - F	100 (60, 160)	120 (80, 190)	160 (90, 290)	30 (17, 40)	70 (50, 110)	NA	80 (60, 120)	20 (17, 30)	50 (30, 80)	840 (530, 1300)
	Adult - M	190 (110, 320)	140 (90, 210)	150 (70, 300)	70 (40, 120)	140 (90, 220)	110 (60, 190)	NA	20 (18, 20)	NA	1100 (760, 1600)
	Child - F	5 (3, 9)	5 (3, 9)	12 (7, 20)	3 (2, 5)	5 (3, 8)	NA	5 (4, 8)	1 (1, 2)	3 (2, 5)	60 (30, 110)
	Child - M	11 (6, 20)	7 (4, 12)	19 (9, 40)	14 (8, 20)	17 (10, 30)	7 (3, 14)	NA	2 (1, 2)	NA	110 (70, 180)
Head and Neck Combined	Adult - F	8 (4, 16)	12 (7, 20)	90 (50, 150)	14 (9, 20)	40 (20, 60)	NA	5 (3, 9)	2 (1, 2)	3 (3, 4)	410 (240, 680)
	Adult - M	16 (7, 40)	12 (7, 20)	140 (70, 280)	60 (40, 100)	70 (40, 100)	11 (7, 19)	NA	2 (2, 2)	NA	500 (320, 790)
Cardiac	Adult - F	30 (16, 50)	40 (30, 70)	8 (4, 14)	0 (0, 0)	19 (12, 30)	NA	4 (2, 6)	1 (1, 1)	2 (2, 3)	160 (110, 240)
	Adult - M	60 (30, 100)	50 (30, 80)	12 (7, 20)	2 (1, 3)	70 (50, 100)	8 (4, 14)	NA	2 (1, 2)	NA	370 (280, 500)
	Child - F	1 (1, 3)	2 (1, 3)	1 (0, 1)	0 (0, 0)	1 (0, 2)	NA	0 (0, 0)	0 (0, 0)	0 (0, 0)	8 (4, 16)
	Child - M	2 (1, 5)	1 (1, 3)	1 (0, 2)	0 (0, 0)	2 (1, 3)	0 (0, 0)	NA	0 (0, 0)	NA	11 (7, 19)
Full Body	Adult - F	100 (50, 200)	130 (70, 230)	16 (8, 30)	1 (1, 2)	30 (20, 40)	NA	70 (40, 110)	19 (14, 30)	40 (20, 70)	390 (220, 690)
	Adult - M	220 (110, 470)	170 (90, 300)	11 (6, 20)	3 (2, 4)	90 (60, 140)	100 (40, 240)	NA	20 (16, 30)	NA	750 (480, 1200)
	Child - F	3 (1, 8)	3 (1, 7)	1 (0, 1)	0 (0, 0)	1 (0, 2)	NA	3 (1, 5)	1 (0, 1)	2 (1, 4)	13 (6, 30)
	Child - M	10 (4, 30)	6 (3, 13)	1 (1, 3)	1 (0, 1)	4 (2, 8)	5 (2, 19)	NA	1 (1, 2)	NA	40 (20, 60)
Extremity	Adult - F	0 (0, 1)	0 (0, 1)	0 (0, 0)	0 (0, 0)	0 (0, 0)	NA	1 (1, 2)	1 (0, 1)	1 (1, 1)	7 (4, 12)
	Adult - M	1 (0, 2)	1 (0, 1)	0 (0, 1)	0 (0, 1)	0 (0, 1)	3 (3, 4)	NA	1 (0, 1)	NA	15 (10, 20)
	Child - F	0 (0, 0)	0 (0, 0)	0 (0, 0)	0 (0, 0)	0 (0, 0)	NA	0 (0, 0)	0 (0, 0)	0 (0, 0)	1 (0, 2)
	Child - M	0 (0, 0)	0 (0, 0)	0 (0, 0)	0 (0, 1)	0 (0, 0)	0 (0, 0)	NA	0 (0, 0)	NA	1 (1, 2)

eTable 3 footnotes:
Exam counts do not sum exactly to the "All" row totals, due to rounding.
NA = not applicable, meaning this category does not exist in this age group.
Dashes (-) mean fewer than 12 exams occurred in this category in the UCSF CT registry; thus it was not included in calculations.

© 2025 Smith-Bindman R et al. *JAMA Internal Medicine.*

eTable 4. Projected number of future cancers (with uncertainty limits, UL) overall and by cancer type that could result from computed tomography exams performed in the U.S. in 2023, by age and sex.

Sex, Age	Total Projected Cancers (UL)	Cancer Type (UL)							
		Lung	Colon	Leukemia	Bladder	Stomach	Thyroid	Breast	Liver
All	102700 (96400, 109500)	22400 (20200, 25000)	8700 (7800, 9700)	7900 (6700, 9500)	7100 (6000, 8500)	7100 (5500, 9100)	7000 (5400, 9200)	5700 (5000, 6500)	4100 (3400, 5000)
Child	9700 (8100, 11600)	990 (870, 1100)	330 (280, 390)	550 (380, 820)	250 (200, 320)	280 (200, 400)	3500 (2300, 5500)	630 (550, 730)	160 (130, 200)
Adult	93000 (86900, 99600)	21400 (19200, 24000)	8400 (7500, 9400)	7400 (6100, 8900)	6900 (5700, 8200)	6800 (5200, 8800)	3500 (2700, 4600)	5100 (4400, 5900)	4000 (3200, 4900)
**Female**									
<1	1900 (860, 4000)	130 (80, 220)	5 (4, 8)	70 (13, 350)	4 (2, 7)	10 (4, 20)	1400 (490,4000)	70 (50, 90)	4 (2, 10)
1-4	1200 (780, 1800)	120 (90, 170)	19 (12, 30)	60 (19, 200)	18 (11, 30)	30 (10, 60)	580 (280, 1200)	140 (110, 190)	9 (4, 20)
5-9	760 (560, 1000)	100 (70, 140)	19 (11, 30)	30 (13, 70)	19 (9, 40)	20 (9, 60)	300 (170, 520)	120 (90, 150)	8 (4, 19)
10-14	890 (680, 1200)	130 (90, 180)	30 (18, 60)	30 (15, 50)	30 (14, 60)	40 (12, 100)	280 (160, 490)	140 (100, 180)	12 (5, 30)
15-17	1100 (830, 1400)	180 (120, 260)	50 (30, 80)	30 (19, 60)	40 (20, 90)	50 (18, 150)	270 (160, 480)	170 (120, 240)	18 (7, 50)
18-29	9200 (7200, 11800)	1800 (1200, 2600)	520 300, 890)	280 (170, 480)	450 (220, 950)	550 (200, 1600)	1600 (930, 2700)	1700 (1300, 2400)	200 (80, 490)
30-39	8200 (6400, 10500)	2000 (1300, 3000)	540 (320, 910)	330 (190, 560)	480 (230, 990)	570 (210, 1500)	680 (410, 1100)	1300 (980, 1800)	210 (90, 500)
40-49	8900 (7000, 11200)	2600 (1800, 3800)	650 (410, 1000)	440 (250, 770)	600 (310, 1200)	700 (280, 1700)	300 (180, 490)	920 (700, 1200)	270 (130, 590)
50-59	10400 (8200, 13000)	3500 (2500, 5000)	760 (490, 1200)	650 (350, 1200)	710 (390, 1300)	830 (350, 2000)	140 (90, 240)	660 (510, 870)	330 (160, 720)
60-69	9100 (7300, 11400)	3300 (2400, 4500)	670 (450, 1000)	790 (400, 1500)	630 (370, 1100)	750 (320, 1800)	40 (20, 70)	330 (250, 430)	310 (140, 680)
70-79	4500 (3600, 5500)	1600 (1200, 2200)	310 (210, 460)	600 (290, 1200)	300 (180, 500)	360 (150, 870)	6 (4, 11)	90 (70, 110)	150 (60, 360)
80-89	820 (640, 1100)	310 (220, 440)	40 (30, 70)	180 (80, 380)	50 (30, 90)	60 (20, 150)	0 (0, 1)	8 (6, 11)	30 (11, 70)
90-99	40 (30, 50)	14 (8, 20)	2 (1, 3)	14 (6, 30)	2 (1, 5)	2 (1, 7)	0 (0, 0)	0 (0, 0)	1 (0, 3)
**Total**	**56900 (51900, 62300)**	**15900 (13800, 18300)**	**3600 (3000, 4300)**	**3500 (2700, 4500)**	**3300 (2600, 4300)**	**4000 (2800, 5700)**	**5600 (4100, 7700)**	**5700 (5000, 6500)**	**1600 (1100, 2100)**

© 2025 Smith-Bindman R et al. *JAMA Internal Medicine.*

eTable 4 (continued: from previous page, projected number of cancers for most common cancer types)

Sex, Age	Total Projected Cancers (UL)	Cancer Type (UL)							
		Lung	Colon	Leukemia	Bladder	Stomach	Thyroid	Breast	Liver
**Male**									
<1	890 (550, 1400)	70 (40, 130)	13 (9, 19)	90 (20, 340)	8 (4, 14)	12 (5, 30)	320 (120, 870)	NA	11 (6, 20)
1-4	870 (600, 1300)	70 (50, 90)	30 (20, 50)	100 (30, 270)	20 (11, 40)	30 (10, 60)	170 (80, 370)	NA	20 (11, 40)
5-9	670 (490, 920)	60 (40, 80)	40 (30, 60)	60 30, 110)	30 (13, 60)	30 (9, 80)	90 (50, 160)	NA	20 (10, 40)
10-14	710 (530, 950)	60 (50, 90)	50 (30, 80)	50 (30, 80)	30 (15, 70)	30 (11, 90)	70 (40, 130)	NA	20 (13, 50)
15-17	780 (610, 1000)	80 (50, 100)	70 (50, 100)	50 (30, 70)	50 (20, 90)	40 (16, 110)	60 (30, 110)	NA	30 (18, 60)
18-29	6400 (5100, 8000)	750 (560, 1000)	700 (490, 990)	360 (240, 520)	420 (220, 780)	410 (170, 990)	430 (260, 690)	NA	330 (190, 570)
30-39	5900 (4700, 7400)	760 (560, 1000)	750 (540, 1000)	370 (250, 560)	460 (250, 830)	420 (180, 990)	170 (110, 270)	NA	350 (210, 600)
40-49	6500 (5100, 8400)	880 (620, 1200)	830 (600, 1200)	460 (290, 740)	570 (300, 1100)	470 (190, 1200)	70 (40, 120)	NA	410 (230, 710)
50-59	9300 (7500, 11700)	1400 (1000, 2000)	1100 (820, 1500)	800 (480, 1400)	860 (490, 1500)	670 (290, 1600)	40 (30, 70)	NA	580 (320, 1000)
60-69	9100 (7400, 11200)	1500 (1100, 2200)	1100 (770, 1400)	1100 (600, 2100)	900 (510, 1600)	660 (280, 1600)	16 (9, 30)	NA	540 (270, 1100)
70-79	4100 (3300, 5100)	740 (490, 1100)	420 (310, 580)	800 (390, 1600)	400 (230, 690)	290 (120, 710)	2 (1, 4)	NA	240 (110, 520)
80-89	600 (460, 780)	110 (70, 190)	40 (30, 70)	200 (90, 410)	50 (20, 90)	40 (14, 100)	0 (0, 0)	NA	30 (13, 80)
90-99	17 (12, 20)	3 (1, 6)	1 (0, 1)	9 (4, 19)	1 (0, 3)	1 (0, 3)	0 (0, 0)	NA	1 (0, 2)
**Total**	**45900 (42100, 50000)**	**6500 (5700, 7500)**	**5100 (4500, 5800)**	**4500 (3500, 5700)**	**3800 (3000, 4800)**	**3100 (2200, 4400)**	**1400 (1100, 1900)**	**NA**	**2600 (2000, 3300)**

© 2025 Smith-Bindman R et al. *JAMA Internal Medicine.*

eTable 4 (continued: projected number of cancers for other cancer types)

Sex, Age	Total Projected Cancers (UL)	Cancer Type (UL)									
		Kidney	Pancreas	Oral Cavity / Pharynx	Brain / CNS	Esophagus	Prostate	Ovary	Rectum	Uterus	Other and ill-defined sites
All	102700 (96400, 109500)	3000 (2300, 3900)	2800 2300, 3500)	2800 (2300, 3400)	1600 (1300, 2000)	1500 (1300, 1800)	1500 (820, 2700)	890 (670, 1200)	560 (480, 660)	550 (400, 760)	17400 (15300, 19800)
Child	9700 (8100, 11600)	130 (90, 180)	100 (80, 140)	450 (310, 650)	440 (320, 620)	110 (90, 150)	70 (30, 170)	40 (30, 70)	30 (20, 40)	30 (16, 50)	1600 (1200, 2000)
Adult	93000 (86900, 99600)	2900 (2200, 3700)	2700 (2200, 3400)	2300 (1900, 2900)	1200 (910, 1500)	1400 (1200, 1700)	1400 (760, 2700)	850 (630, 1100)	540 (450, 630)	530 (380, 730)	15800 (13700, 18200)
**Female**											
<1	1900 (860, 4000)	3 (1, 5)	3 (1, 5)	40 (15, 100)	17 (7, 50)	6 (2, 20)	NA	2 (1, 3)	0 (0, 1)	1 (0, 3)	110 (40, 350)
1-4	1200 (780, 1800)	7 (3, 16)	8 (4, 15)	50 (18, 120)	19 (8, 50)	6 (3, 11)	NA	7 (4, 14)	2 (1, 5)	5 (2, 13)	120 (50, 280)
5-9	760 (560, 1000)	7 (3, 17)	7 (3, 16)	30 (12, 70)	11 (5, 30)	4 (2, 6)	NA	7 (3, 16)	2 (1, 5)	5 (1, 14)	80 (40, 170)
10-14	890 (680, 1200)	12 (4, 30)	12 (5, 30)	40 (15, 90)	14 (6, 30)	5 (3, 7)	NA	11 (5, 30)	3 (1, 7)	7 (2, 20)	110 (60, 220)
15-17	1100 (830, 1400)	19 (7, 50)	19 (8, 50)	40 (15, 90)	15 7, 30)	6 (4, 9)	NA	16 (7, 40)	5 (2, 9)	10 (4, 30)	140 (70, 260)
18-29	9200 (7200, 11800)	210 (70, 580)	200 (80, 470)	220 (100, 450)	60 (30, 120)	60 (40, 90)	NA	150 (70, 340)	50 (20, 90)	90 (40, 250)	1100 (580, 2000)
30-39	8200 (6400, 10500)	210 (80, 570)	210 (90, 490)	200 (90, 440)	50 (20, 100)	60 (40, 100)	NA	150 (70, 320)	40 (30, 80)	90 (40, 210)	1100 570, 1900)
40-49	8900 (7000, 11200)	250 (100, 590)	270 (130, 580)	200 (90, 450)	50 (20, 100)	80 (50, 120)	NA	170 (80, 340)	50 (30, 80)	100 (50, 220)	1200 (670, 2100)
50-59	10400 (8200, 13000)	250 (110, 560)	320 (160, 650)	270 (140, 550)	50 (30, 100)	100 (70, 150)	NA	180 (90, 350)	50 (30, 80)	110 (50, 230)	1400 (830, 2400)
60-69	9100 (7300, 11400)	190 (90, 410)	280 (150, 560)	220 (110, 460)	40 (19, 80)	90 (60, 120)	NA	140 (70, 260)	40 (30, 60)	90 (50, 160)	1200 (700, 2000)
70-79	4500 (3600, 5500)	70 (40, 150)	130 (70, 240)	100 (50, 190)	15 (7, 30)	40 (30, 50)	NA	60 (30, 120)	19 (15, 20)	40 (20, 60)	540 (340, 860)
80-89	820 (640, 1100)	10 (5, 20)	18 (10, 30)	15 (7, 30)	3 (1, 6)	6 (5, 7)	NA	8 (4, 19)	3 (3, 3)	6 (5, 8)	80 (50, 120)
90-99	40 (30, 50)	0 (0, 1)	1 (0, 1)	1 (0, 1)	0 (0, 0)	0 (0, 0)	NA	0 (0, 1)	0 (0, 0)	0 (0, 0)	3 (2, 4)
**Total**	**56900 (51900, 62300)**	**1200 (860, 1800)**	**1500 (1100, 2000)**	**1400 (1100, 1900)**	**350 (270, 440)**	**460 (400, 540)**	**NA**	**890 (670, 1200)**	**270 (220, 330)**	**550 (400, 760)**	**7100 (5700, 8800)**

© 2025 Smith-Bindman R et al. *JAMA Internal Medicine.*

eTable 4 (continued: from previous page, projected number of cancers for other cancer types)

Sex, Age	Total Projected Cancers (UL)	Cancer Type (UL)									
		Kidney	Pancreas	Oral Cavity / Pharynx	Brain / CNS	Esophagus	Prostate	Ovary	Rectum	Uterus	Other and ill-defined sites
**Male**											
<1	890 (550, 1400)	6 (3, 14)	4 (2, 8)	50 (13, 190)	70 (30, 180)	20 (8, 60)	4 (1, 30)	NA	1 (0, 3)	NA	200 (80, 470)
1-4	870 (600, 1300)	13 (6, 30)	9 (4, 18)	70 (18, 240)	90 (40, 230)	20 (11, 50)	11 (2, 60)	NA	3 (1, 7)	NA	220 (120, 440)
5-9	670 (490, 920)	15 (5, 40)	10 (4, 20)	50 (14, 170)	70 (30, 150)	14 (8, 20)	15 (2, 90)	NA	3 (1, 9)	NA	180 (100, 330)
10-14	710 (530, 950)	19 (7, 50)	12 (6, 30)	50 (17, 160)	70 (30, 160)	14 (9, 20)	19 (3, 120)	NA	4 (2, 11)	NA	200 (120, 330)
15-17	780 (610, 1000)	30 (11, 70)	18 (9, 40)	50 (17, 130)	60 (30, 140)	16 (10, 30)	20 (5, 110)	NA	5 (3, 12)	NA	210 (130, 320)
18-29	6400 (5100, 8000)	270 (110, 690)	170 (90, 350)	290 (130, 640)	270 (140, 520)	160 (100, 240)	210 (40, 1000)	NA	50 (20, 100)	NA	1600 (1100, 2400)
30-39	5900 (4700, 7400)	290 (120, 710)	190 (100, 370)	190 (90, 410)	150 (80, 270)	150 (90, 230)	240 (50, 1100)	NA	50 (20, 90)	NA	1400 (910, 2100)
40-49	6500 (5100, 8400)	310 (120, 790)	220 (110, 440)	190 (90, 400)	140 (70, 280)	160 (100, 260)	300 (60, 1400)	NA	50 (30, 100)	NA	1500 (950, 2300)
50-59	9300 (7500, 11700)	390 (170, 890)	310 (160, 580)	220 (100, 470)	160 (80, 330)	240 (150, 380)	370 (90, 1500)	NA	60 (40, 110)	NA	2100 (1400, 3200)
60-69	9100 (7400, 11200)	310 (140, 700)	280 (150, 530)	150 (70, 330)	120 (60, 250)	210 (120, 350)	240 (70, 800)	NA	50 (30, 80)	NA	1900 (1300, 2900)
70-79	4100 (3300, 5100)	100 (50, 190)	110 (60, 190)	50 (20, 100)	40 (19, 80)	80 (50, 140)	60 (30, 130)	NA	20 (15, 30)	NA	770 (530, 1100)
80-89	600 (460, 780)	10 (5, 19)	11 (6, 20)	6 (3, 13)	5 (2, 10)	9 (5, 17)	6 (4, 8)	NA	2 (2, 3)	NA	80 (60, 120)
90-99	17 (12, 20)	0 (0, 0)	0 (0, 0)	0 (0, 0)	0 (0, 0)	0 (0, 0)	0 (0, 0)	NA	0 (0, 0)	NA	2 (1, 2)
**Male**	**45900 (42100, 50000)**	**1700 (1200, 2500)**	**1300 (1000, 1800)**	**1400 (1000, 1800)**	**1300 (990, 1600)**	**1100 (890, 1300)**	**1500 (820, 2700)**	**NA**	**300 (230, 380)**	**NA**	**10300 (8800, 12100)**

eTable 4 footnote:
NA = not applicable, meaning this cancer type was not calculated for this sex.

© 2025 Smith-Bindman R et al. *JAMA Internal Medicine.*

eTable 5. Projected number of future cancers (with uncertainty limits, UL) by CT category, age and sex that could result from computed tomography exams performed in the U.S. in 2023.

Age	Total Projected Cancers (UL)	Projected Cancers (UL) by Body Region and CT Category												
		Abdomen			Head			Chest		Spine				
		Low Dose	Routine Dose	High Dose	Low Dose	Routine Dose	High Dose	Low Dose	Routine Dose	Cervical	Thoracic or Lumbar	Thoracic & Lumbar	Thoracic	Lumbar
All	102700 (96400, 109500)	1300 (1100, 1600)	32800 (28300, 37900)	5000 (4300, 5800)	1900 (1600, 2200)	10200 (8300, 12500)	420 (340, 520)	40 (30, 50)	22700 (19500, 26300)	9600 (8300, 11100)	3000 (2600, 3400)	180 (150, 230)	90 (70, 110)	60 (50, 80)
Child	9700 (8100, 11600)	NA	1600 (1300, 1900)	40 (30, 50)	460 (360, 580)	4700 (3300, 6600)	5 (3, 8)	NA	1200 (960, 1400)	1100 (900, 1400)	NA	NA	90 (70, 110)	60 (50, 80)
Adult	93000 (86900, 99600)	1300 (1100, 1600)	31200 (26800, 36300)	5000 (4300, 5800)	1400 (1100, 1800)	5500 (4400, 6900)	420 (330, 520)	40 (30, 50)	21500 (18400, 25100)	8500 (7200, 10000)	3000 (2600, 3400)	180 (150, 230)	NA	NA
**Female**														
<1	1900 (860, 4000)	NA	14 (8, 20)	--	30 (12, 60)	1600 (680, 3900)	--	NA	120 (60, 230)	40 (19, 70)	NA	NA	--	--
1-4	1200 (780, 1800)	NA	120 (70, 200)	--	60 (20, 140)	560 (240, 1300)	--	NA	240 (130, 440)	170 (90, 320)	NA	NA	--	--
5-9	760 (560, 1000)	NA	160 (90, 260)	--	50 (20, 110)	220 (100, 470)	--	NA	110 (60, 210)	160 (80, 320)	NA	NA	4 (3, 8)	4 (2, 7)
10-14	890 (680, 1200)	NA	250 (150, 410)	6 (4, 10)	50 (20, 120)	200 (100, 420)	--	NA	130 (70, 240)	170 (80, 330)	NA	NA	30 (16, 40)	10 (6, 18)
15-17	1100 (830, 1400)	NA	370 (230, 610)	8 (5, 13)	50 (20, 110)	160 (80, 340)	--	NA	180 (100, 330)	180 (90, 370)	NA	NA	19 (11, 30)	18 (11, 30)
18-29	9200 (7200, 11800)	140 (90, 240)	3700 (2200, 6000)	190 (120, 300)	220 (110, 450)	690 (340, 1400)	18 (9, 40)	--	1700 (990, 3000)	1400 (780, 2500)	190 (120, 300)	--	NA	NA
30-39	8200 (6400, 10500)	160 (90, 260)	3400 (2100, 5500)	190 (130, 300)	120 (60, 250)	480 (230, 1000)	40 (19, 80)	--	1900 (1100, 3300)	910 (530, 1600)	130 (80, 200)	--	NA	NA
40-49	8900 (7000, 11200)	150 (90, 250)	3300 (2000, 5400)	360 (240, 550)	100 (50, 210)	450 (210, 980)	50 (20, 100)	--	2000 (1100, 3500)	870 (490, 1500)	250 (160, 380)	--	NA	NA
50-59	10400 (8200, 13000)	120 (70, 200)	3400 (2100, 5400)	540 (350, 840)	90 (40, 200)	470 (210, 1100)	50 (20, 100)	9 (5, 17)	2800 (1600, 4800)	780 (440, 1400)	320 (200, 510)	60 (40, 90)	NA	NA
60-69	9100 (7300, 11400)	90 (50, 140)	2500 (1500, 4000)	490 (320, 760)	60 (30, 130)	440 (200, 950)	30 (13, 60)	11 (6, 19)	2800 (1700, 4700)	750 (440, 1300)	360 (230, 580)	30 (19, 50)	NA	NA
70-79	4500 (3600, 5500)	20 (15, 40)	1100 (650, 1800)	240 (150, 380)	30 (13, 50)	230 (100, 490)	20 (9, 40)	5 (3, 8)	1300 (790, 2100)	380 (230, 620)	190 (110, 310)	17 (11, 30)	NA	NA
80-89	820 (640, 1100)	4 (2, 8)	210 (110, 400)	40 (20, 70)	6 (3, 12)	50 (20, 130)	5 (2, 10)	--	180 (100, 350)	70 (40, 120)	30 (17, 60)	5 (3, 9)	NA	NA
90-99	40 (30, 50)	--	11 (5, 20)	2 (1, 5)	1 (0, 2)	4 (1, 10)	0 (0, 1)	--	7 (3, 16)	5 (2, 11)	2 (1, 3)	--	NA	NA
Total Female	56900 (51900, 62300)	690 (550, 860)	18400 (15100, 22400)	2100 (1700, 2500)	870 (670, 1100)	5600 (4100, 7700)	210 (150, 280)	30 (18, 40)	13500 (10900, 16700)	5900 (4700, 7300)	1500 (1200, 1800)	110 (90, 150)	50 (40, 70)	30 (20, 50)

© 2025 Smith-Bindman R et al. *JAMA Internal Medicine.*

eTable 5 (continued: from previous page)

Age	Total Projected Cancers (UL)	Projected Cancers (UL) by Body Region and CT Category												
		Abdomen			Head			Chest		Spine				
		Low Dose	Routine Dose	High Dose	Low Dose	Routine Dose	High Dose	Low Dose	Routine Dose	Cervical	Thoracic or Lumbar	Thoracic & Lumbar	Thoracic	Lumbar
**Male**														
<1	890 (550, 1400)	NA	30 (14, 50)	--	30 (15, 50)	720 (400, 1300)	--	NA	70 (40, 120)	18 (11, 30)	NA	NA	--	--
1-4	870 (600, 1300)	NA	100 (60, 180)	5 (3, 8)	40 (20, 70)	490 (260, 930)	--	NA	90 (60, 150)	100 (60, 160)	NA	NA	--	--
5-9	670 (490, 920)	NA	160 (90, 290)	3 (2, 5)	40 (20, 90)	270 (150, 520)	--	NA	70 (40, 110)	80 (50, 130)	NA	NA	5 (3, 8)	5 (3, 8)
10-14	710 (530, 950)	NA	180 (100, 340)	4 (2, 6)	60 (30, 110)	240 (120, 470)	--	NA	80 (50, 130)	90 (50, 150)	NA	NA	14 (8, 20)	7 (4, 11)
15-17	780 (610, 1000)	NA	200 (110, 380)	13 (8, 20)	50 (30, 100)	180 (90, 360)	5 (3, 8)	NA	90 (50, 150)	120 (80, 200)	NA	NA	17 (10, 30)	18 (11, 30)
18-29	6400 (5100, 8000)	110 (60, 210)	2100 (1200, 3600)	170 (100, 300)	270 (140, 500)	710 (360, 1400)	30 (16, 60)	--	780 (480, 1300)	830 (520, 1300)	240 (160, 380)	--	NA	NA
30-39	5900 (4700, 7400)	110 (60, 200)	2100 (1200, 3700)	260 (160, 440)	190 (100, 360)	380 (190, 780)	30 (14, 60)	--	1000 (630, 1600)	500 (310, 800)	280 (180, 430)	--	NA	NA
40-49	6500 (5100, 8400)	140 (80, 260)	2500 (1400, 4400)	330 (180, 580)	100 (50, 220)	410 (190, 870)	30 (16, 70)	--	1300 (810, 2100)	500 (310, 820)	200 (130, 310)	--	NA	NA
50-59	9300 (7500, 11700)	150 (90, 270)	2900 (1700, 5200)	820 (500, 1300)	110 (50, 220)	510 (240, 1100)	40 (19, 90)	3 (2, 5)	2200 (1400, 3400)	640 (400, 1000)	340 (220, 510)	--	NA	NA
60-69	9100 (7400, 11200)	100 (60, 170)	2800 (1700, 4500)	860 (540, 1400)	70 (40, 140)	470 (220, 1000)	50 (20, 100)	8 (5, 12)	2300 (1400, 3800)	550 (340, 900)	280 (190, 430)	50 (30, 70)	NA	NA
70-79	4100 (3300, 5100)	30 (20, 60)	1100 (700, 1900)	410 (250, 660)	30 (12, 50)	190 (80, 430)	20 (10, 50)	4 (3, 7)	1000 (610, 1800)	220 (130, 380)	140 (90, 220)	20 (15, 40)	NA	NA
80-89	600 (460, 780)	4 (2, 8)	150 (80, 300)	50 (30, 100)	5 (2, 12)	40 (14, 100)	2 (1, 5)	--	140 (70, 280)	40 (20, 80)	20 (13, 40)	--	NA	NA
90-99	17 (12, 20)	--	5 (2, 11)	--	0 (0, 1)	2 (1, 6)	--	--	4 (2, 8)	2 (1, 5)	1 (0, 2)	--	NA	NA
Total Male	45900 (42100, 50000)	650 (510, 840)	14400 (11600, 17900)	2900 (2300, 3700)	990 (770, 1300)	4600 (3700, 5800)	210 (160, 290)	15 (11, 20)	9200 (7500, 11200)	3700 (3100, 4400)	1500 (1300, 1800)	70 (50, 100)	40 (30, 50)	30 (20, 40)

© 2025 Smith-Bindman R et al. *JAMA Internal Medicine.*

eTable 5 (continued: with additional CT categories)

Age	Total Projected Cancers (UL)	Projected Cancers (UL) by Body Region and CT Category									
		Head and Neck Combined		Cardiac		Chest/ Cardiac High Dose	Full Body[a]	Extremity			
		Routine Dose	High Dose	Low Dose	Routine Dose			All Adults	Upper, Routine Dose	Lower, Routine Dose	Lower, High Dose
All	102700 (96400, 109500)	3900 (3300, 4600)	200 (150, 260)	310 (250, 390)	1300 (1100, 1400)	1800 (1600, 2100)	8000 (7000, 9100)	70 (50, 80)	4 (2, 6)	4 (3, 6)	1 (0, 1)
Child	9700 (8100, 11600)	NA	NA	NA	170 (140, 210)	NA	320 (260, 390)	NA	4 (2, 6)	4 (3, 6)	1 (0, 1)
Adult	93000 (86900, 99600)	3900 (3300, 4600)	200 (150, 260)	310 (250, 390)	1100 (970, 1300)	1800 (1600, 2100)	7600 (6600, 8800)	70 (50, 80)	NA	NA	NA
**Female**											
<1	1900 (860, 4000)	NA	NA	NA	40 (19, 70)	NA	--	NA	--	--	--
1-4	1200 (780, 1800)	NA	NA	NA	13 (7, 20)	NA	40 (20, 70)	NA	--	--	--
5-9	760 (560, 1000)	NA	NA	NA	40 (20, 60)	NA	20 (13, 40)	NA	2 (1, 4)	1 (0, 2)	--
10-14	890 (680, 1200)	NA	NA	NA	20 (12, 40)	NA	20 (14, 40)	NA	1 (0, 2)	1 (1, 2)	0 (0, 0)
15-17	1100 (830, 1400)	NA	NA	NA	14 (8, 20)	NA	70 (40, 110)	NA	0 (0, 0)	1 (0, 1)	0 (0, 0)
18-29	9200 (7200, 11800)	600 (330, 1100)	--	--	--	--	370 (230, 600)	2 (1, 5)	NA	NA	NA
30-39	8200 (6400, 10500)	420 (250, 710)	--	--	--	50 (30, 90)	440 (270, 710)	3 (2, 6)	NA	NA	NA
40-49	8900 (7000, 11200)	390 (220, 670)	--	50 (30, 100)	240 (170, 340)	60 (40, 110)	560 (340, 920)	3 (1, 5)	NA	NA	NA
50-59	10400 (8200, 13000)	430 (250, 760)	60 (40, 110)	70 (40, 130)	180 (120, 250)	110 (70, 180)	910 (550, 1500)	8 (5, 15)	NA	NA	NA
60-69	9100 (7300, 11400)	270 (160, 460)	40 (20, 70)	40 (20, 70)	100 (60, 140)	230 (160, 330)	880 (540, 1400)	8 (4, 14)	NA	NA	NA
70-79	4500 (3600, 5500)	200 (120, 340)	20 (14, 40)	13 (8, 20)	50 (30, 70)	240 (170, 340)	410 (250, 680)	5 (3, 8)	NA	NA	NA
80-89	820 (640, 1100)	60 (30, 120)	--	--	20 (13, 40)	50 (30, 90)	80 (40, 140)	0 (0, 1)	NA	NA	NA
90-99	40 (30, 50)	5 (2, 12)	--	--	--	--	3 (1, 6)	0 (0, 0)	NA	NA	NA
Total Female	56900 (51900, 62300)	2400 (1900, 3000)	120 (90, 180)	180 (130, 250)	700 (590, 840)	750 (620, 910)	3800 (3100, 4700)	30 (20, 40)	3 (1, 5)	3 (2, 4)	0 (0, 0)

© 2025 Smith-Bindman R et al. *JAMA Internal Medicine.*

eTable 5 (continued: from previous page)

Age	Total Projected Cancers (UL)	Projected Cancers (UL) by Body Region and CT Category									
		Head and Neck Combined		Cardiac		Chest/ Cardiac High Dose	Full Body[a]	Extremity			
		Routine Dose	High Dose	Low Dose	Routine Dose			All Adults	Upper, Routine Dose	Lower, Routine Dose	Lower, High Dose
**Male**											
<1	890 (550, 1400)	NA	NA	NA	19 (11, 30)	NA	6 (4, 10)	NA	--	--	--
1-4	870 (600, 1300)	NA	NA	NA	11 (7, 18)	NA	30 (20, 50)	NA	--	--	--
5-9	670 (490, 920)	NA	NA	NA	7 (4, 12)	NA	20 (13, 40)	NA	0 (0, 1)	0 (0, 0)	--
10-14	710 (530, 950)	NA	NA	NA	7 (4, 12)	NA	30 (20, 60)	NA	0 (0, 1)	1 (0, 2)	0 (0, 0)
15-17	780 (610, 1000)	NA	NA	NA	7 (4, 12)	NA	80 (50, 120)	NA	0 (0, 1)	1 (0, 1)	1 (0, 1)
18-29	6400 (5100, 8000)	480 (300, 770)	--	--	90 (60, 140)	100 (70, 150)	480 (300, 770)	5 (2, 12)	NA	NA	NA
30-39	5900 (4700, 7400)	220 (130, 360)	--	12 (7, 20)	120 (80, 160)	110 (70, 160)	590 (380, 910)	10 (5, 20)	NA	NA	NA
40-49	6500 (5100, 8400)	200 (120, 350)	--	30 (17, 50)	--	90 (60, 140)	670 (430, 1000)	7 (3, 14)	NA	NA	NA
50-59	9300 (7500, 11700)	270 (150, 460)	30 (15, 50)	50 (30, 80)	110 (80, 160)	210 (140, 310)	950 (610, 1500)	8 (4, 17)	NA	NA	NA
60-69	9100 (7400, 11200)	190 (110, 320)	30 (19, 60)	30 (16, 50)	140 (100, 200)	300 (190, 460)	870 (560, 1300)	6 (3, 11)	NA	NA	NA
70-79	4100 (3300, 5100)	130 (80, 230)	12 (6, 20)	17 (10, 30)	70 (40, 110)	230 (150, 360)	380 (230, 610)	2 (1, 4)	NA	NA	NA
80-89	600 (460, 780)	30 (13, 50)	3 (1, 6)	--	--	60 (30, 100)	50 (30, 100)	0 (0, 0)	NA	NA	NA
90-99	17 (12, 20)	1 (1, 3)	--	--	--	1 (0, 2)	1 (0, 2)	--	NA	NA	NA
Total Male	45900 (42100, 50000)	1500 (1200, 1900)	70 (50, 100)	130 (100, 170)	580 (490, 670)	1100 (910, 1300)	4200 (3500, 5000)	40 (30, 50)	1 (1, 2)	2 (1, 3)	1 (0, 1)

eTable 5 footnotes:
NA = not applicable, meaning this category does not exist in this age group.
Dashes (-) mean fewer than 12 exams occurred in this category in the UCSF CT registry; thus it was not included in calculations.
[a] Full Body includes Whole Body exams in children and combined Chest-Abdomen-Pelvis exams in adults.

© 2025 Smith-Bindman R et al. *JAMA Internal Medicine.*

eTable 6. Projected number of cancers (with uncertainty limits, UL) in the U.S. for each sensitivity analysis changing the baseline model assumptions.

	Projected Future Cancers	(UL)	Adults		Children		Percent Change from Baseline
			N	%	N	%	
Baseline model cancer projections	102700	(96400, 109500)	93,000	90.6%	9,700	9.4%	0.0%
Use male lung cancer risk coefficients for females	94500	(90000, 99300)	85,200	90.2%	9,400	9.9%	-8.0%
Reduce all imaging volume by 10%	92500	(86800, 98600)	83,700	90.5%	8,700	9.4%	-9.9%
Increase all imaging volume by 10%	113000	(106000, 120500)	102,300	90.5%	10,700	9.5%	10.0%
Reduce all organ doses by 20%	79900	(74900, 85200)	72,300	90.5%	7,600	9.5%	-22.2%
Increase all organ doses by 20%	126600	(118800, 134800)	114,700	90.6%	11,900	9.4%	23.3%
Use IMV estimate of the percentage of all exams that occur in children (9%)	114000	(106700, 121900)	87,500	76.8%	26,500	23.2%	11.0%
Use years 2018-2019 to estimate distribution in CT exams by CT category, age, and sex	103800	(97500, 110400)	94,000	90.6%	9,700	9.3%	1.1%
Exclude exams performed in patients' last 2 years of life	100000	(93700, 106600)	90,300	90.3%	9,600	9.6%	-2.6%

© 2025 Smith-Bindman R et al. *JAMA Internal Medicine.*

eFigure 1. Projected number of CT-induced cancers and 90% uncertainty limits by cancer type in adults (eFigure 1A) and children (eFigure 1B), by sex.

eFigure 1A: Adults

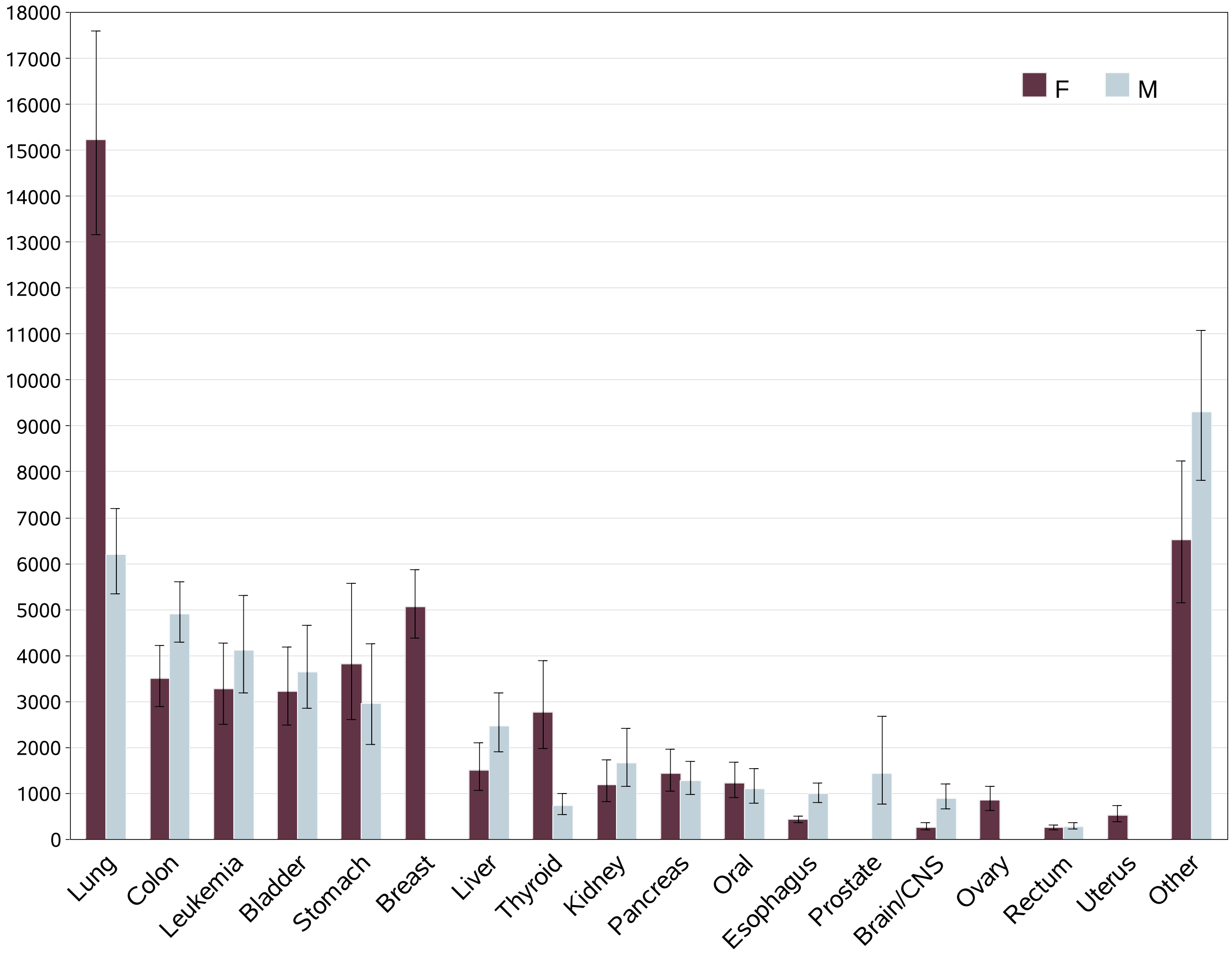

© 2025 Smith-Bindman R et al. *JAMA Internal Medicine.*

eFigure 1B: Children

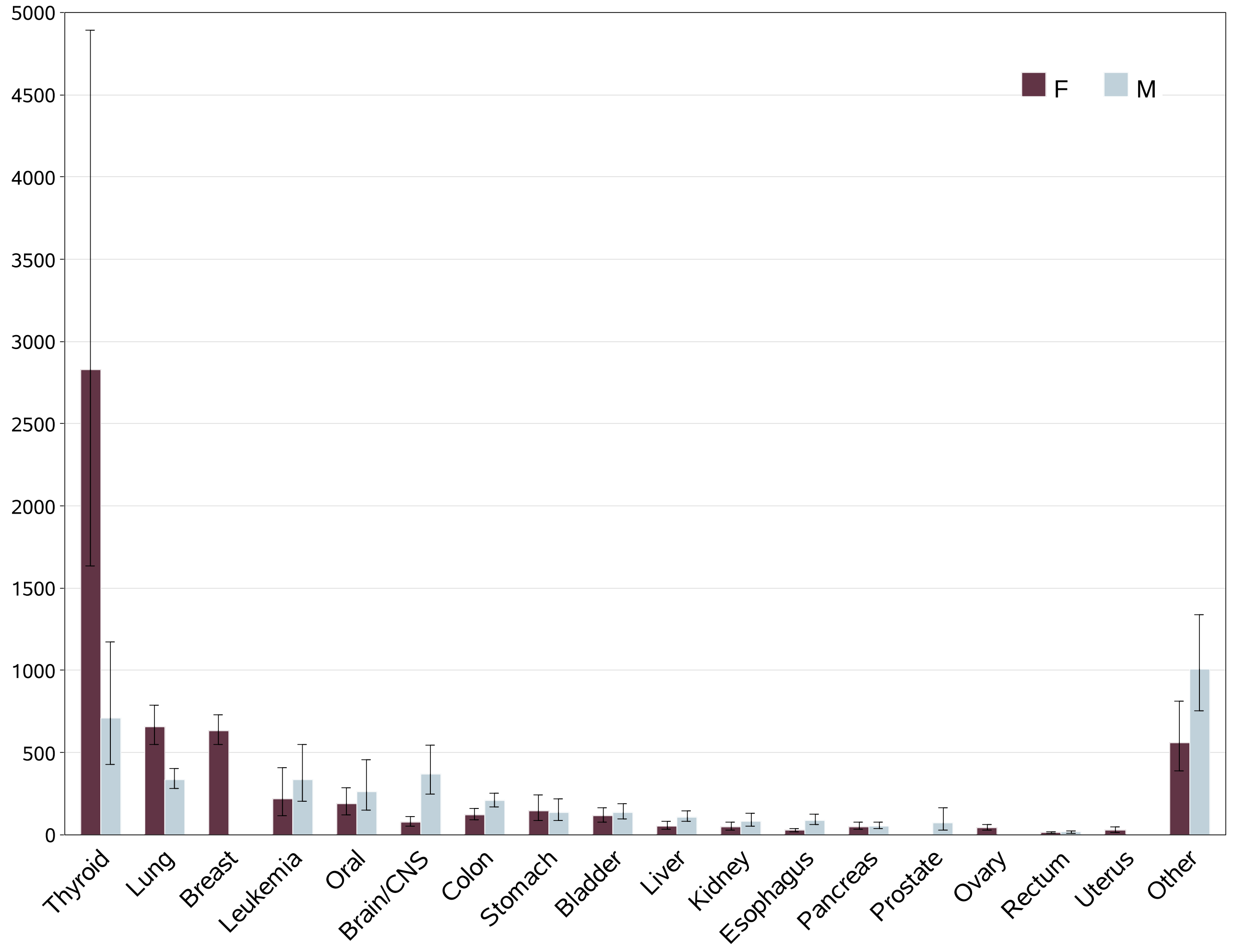

© 2025 Smith-Bindman R et al. *JAMA Internal Medicine.*
