# Supplementary material for: Projected Lifetime Cancer Risks From Current Computed Tomography Imaging
Source: JAMA Intern Med. 2025 Apr 14;185(6):710–9. doi: 10.1001/jamainternmed.2025.0505 (PMC11997853; doi:10.1001/jamainternmed.2025.0505)
Supplement: Supplement 2. — Data Sharing Statement [file jamainternmed-e250505-s002.pdf]

Data Sharing Statement

Smith-Bindman. Projected Lifetime Cancer Risks From Current Computed Tomography Imaging. *JAMA Intern Med*. Published April 14, 2025. doi:10.1001/jamainternmed.2025.0505

Data

Data available: Some data are available upon reasonable request.
